# Genome-wide identification, characterization, and expression analysis of the SOS1 gene family in the medicinal plant *Paeonia ostii* under salt stress

**DOI:** 10.3389/fpls.2025.1614011

**Published:** 2025-07-30

**Authors:** Yahong Chen, Huabing Liang, Zhanchang Wang, Quaid Hussain, Xuexiong Zhang, Fazhi Chen, Xiaohua Wang

**Affiliations:** ^1^ College of Life Sciences, Wuchang University of Technology, Wuhan, Hubei, China; ^2^ Wuhan Academy of Agricultural Sciences, Wuhan, Hubei, China; ^3^ College of Life Sciences and Oceanography, Shenzhen University, Shenzhen, China; ^4^ Baokang County Forestry Bureau, Xiangyang, Hubei, China

**Keywords:** *Paeonia ostii*, SOS1 gene family, salinity stress, miRNAs, expression analysis

## Abstract

*Paeonia ostii* is a commercially important ornamental and traditional medicinal plant esteemed in China. Salt stress is a widespread abiotic stress that significantly affects plant growth and development, and moderate stress can significantly promote the synthesis of plant secondary metabolites, requiring clarification of its underlying molecular mechanisms. The Salt Overly Sensitive 1 (SOS1) gene family is essential for salt stress tolerance, encoding Na^+^/H^+^ antiporters that preserve ion homeostasis and reduce cellular damage. This study conducted an extensive genome-wide analysis of the SOS1 gene family in *Paeonia ostii*, encompassing gene identification, characterization, three-dimensional and secondary structure prediction, gene structure and motif analysis, multiple alignments, phylogenetic tree construction, chromosomal localization, cis-regulatory element analysis, synteny analysis, Ka/Ks calculation, and gene expression analysis under salt stress treatments in three cultivars. Our findings identified 19 *SOS1* genes within the *P. ostii* genome, demonstrating unique structural and functional attributes. All *SOS1* genes were located on the plasma membrane and distributed across five chromosomes and two scaffolds. The conserved motif analysis results indicated that the SOS1 homologs had comparable protein structures. The coding sections of 19 *PoSOS1* genes comprise amino acid sequences varying from 455 to 859, whereas the exons encompass amino acids ranging from 3 to 20. Furthermore, we discovered that the 2.5 kb upstream promoter region of the *PoSOS1s* gene has many cis-elements linked to phytohormones and stress responses. The phylogenetic study categorized the *PoSOS1* genes into three subfamilies. In total, 38 miRNAs that target 19 *PoSOS1* genes from 18 distinct families were identified. Conversely, gene expression analysis revealed six differentially expressed *SOS1* genes in three distinct cultivars subjected to salt stress, with all six genes down-regulated and only one gene up-regulated in the QF-230 cultivar after six days of salt stress. This study offers new insights into the *SOS1* gene family in *P. ostii*, elucidating its function in salt stress tolerance and establishing a foundation for future research on the functional characterization of *SOS1* genes in *P. ostii*.

## 1 Introduction

Over 800 million hectares of arable land worldwide are affected by soil salinization and alkalization. In China, saline-alkali soils constitute 25% of the total arable land area but remain underutilized (Liu and Wang, 2021). Plants are susceptible to adverse environmental conditions due to their sessile nature (Hussain et al., 2021). However, their survival in natural habitats depends on adaptations to both abiotic and biotic stressors (Shang et al., 2024). Salinity hinders plant growth and development through water deficit, ionic toxicity (primarily Na^+^ and Cl^-^ accumulation), and nutrient imbalances. In salinity-affected regions, crop yields have been reported to decrease by 20–50% (Isayenkov and Maathuis, 2019; Isayenkov, 2012; Muhammad et al., 2024; Eswar et al., 2021). Besides, former research had demonstrated that moderate stress could significantly promote the synthesis of plant secondary metabolites; the expressions of *PAL*, *4CL*, *C4H*, and *COMT* genes and their protein contents were up-regulated under low salt stress, which was positively correlated with the relative contents of phenolic acid and flavonoid, chlorogenic acid and luteolin (Cai et al., 2020, 2021; Khare et al., 2020).

The Salt Overly Sensitive (SOS) signaling pathway, critical for plant salt-stress responses, consists of three core components: *SOS1*, *SOS2*, and *SOS3* (Liang et al., 2023; Cheng et al., 2019). *SOS1* is a plasma membrane-localized Na^+^/H^+^ antiporter that mediates Na^+^ efflux from root cells and xylem loading to facilitate long-distance ion transport (Świeżawska et al., 2018; Shi et al., 2000). The *SOS1* homologs in *Arabidopsis thaliana* were originally designated as *AtNHX1* to *AtNHX8*. *AtSOS1* exhibits tissue-specific expression patterns, with high abundance in root epidermal cells, xylem parenchyma, and vascular tissues, indicating its dual role in rhizosphere Na^+^ exclusion and systemic ion homeostasis (Ali et al., 2021; Keisham et al., 2018; Liang et al., 2023; Gao et al., 2016).

*Paeonia* sect. Moutan, commonly known as the tree peony or “King of Flowers” in China, belongs to the *Paeoniaceae* family. This culturally significant species has been cultivated for over 1,500 years, with documented uses in traditional medicine, ornamental horticulture, and oil production (Zhang et al., 2017, 2014). Officially recognized as an industrial oil crop by Chinese authorities, *P. ostii* demonstrates exceptional adaptability to marginal lands, with cultivation spanning over 20 provinces due to its high seed yield (greater than 20% oil content), drought tolerance, and nutritional value (Li et al., 2015; Zhao et al., 2021). Soil salinization has impeded the advancement of the oil tree peony business. It has led to a diminished emergence rate, protracted development, and even mortality of seedlings, reducing crop yield and quality and resulting in significant economic losses for cultivators (Xiong et al., 2019). Consequently, it is essential to investigate the enhancement of tree peony salt tolerance, which can offer theoretical support for the sustainable advancement of the tree peony business (Shi et al., 2023). Investigating the characteristics of salt-tolerant gene families in *Paeonia ostii* (a traditional Chinese medicinal herb) holds significant potential for developing novel salt-tolerant cultivars. Such advancements would enhance the utilization value of saline-alkali lands, expand the cultivation range of *Paeonia ostii*, and provide additional land resources for cultivating this traditional medicinal herb.

Despite extensive characterization of *SOS1* in model plants, this gene family remains uncharacterized in *Paeonia ostii*. Thus, this research represents the initial attempt to perform a genome-wide analysis to identify *SOS1* genes within the genome of *P. ostii*. This study aimed to identify and characterize 19 *SOS1* genes and then investigate their expression levels in three cultivars, QF-11, QF-12, and QF-230, and their response to salt treatments. We employed comparative genomics and phylogenomic approaches to investigate the evolutionary trajectory, structural diversification, and stress-responsive expression patterns of *SOS1* genes in *P. ostii*. This study provides a foundational framework for molecular breeding programs to enhance salt tolerance in *P. ostii*, while elucidating conserved and lineage-specific adaptations in SOS-mediated ion homeostasis.

## 2 Results

### 2.1 Identification of the PoSOS1s family and its physicochemical properties

**Figure 1:**
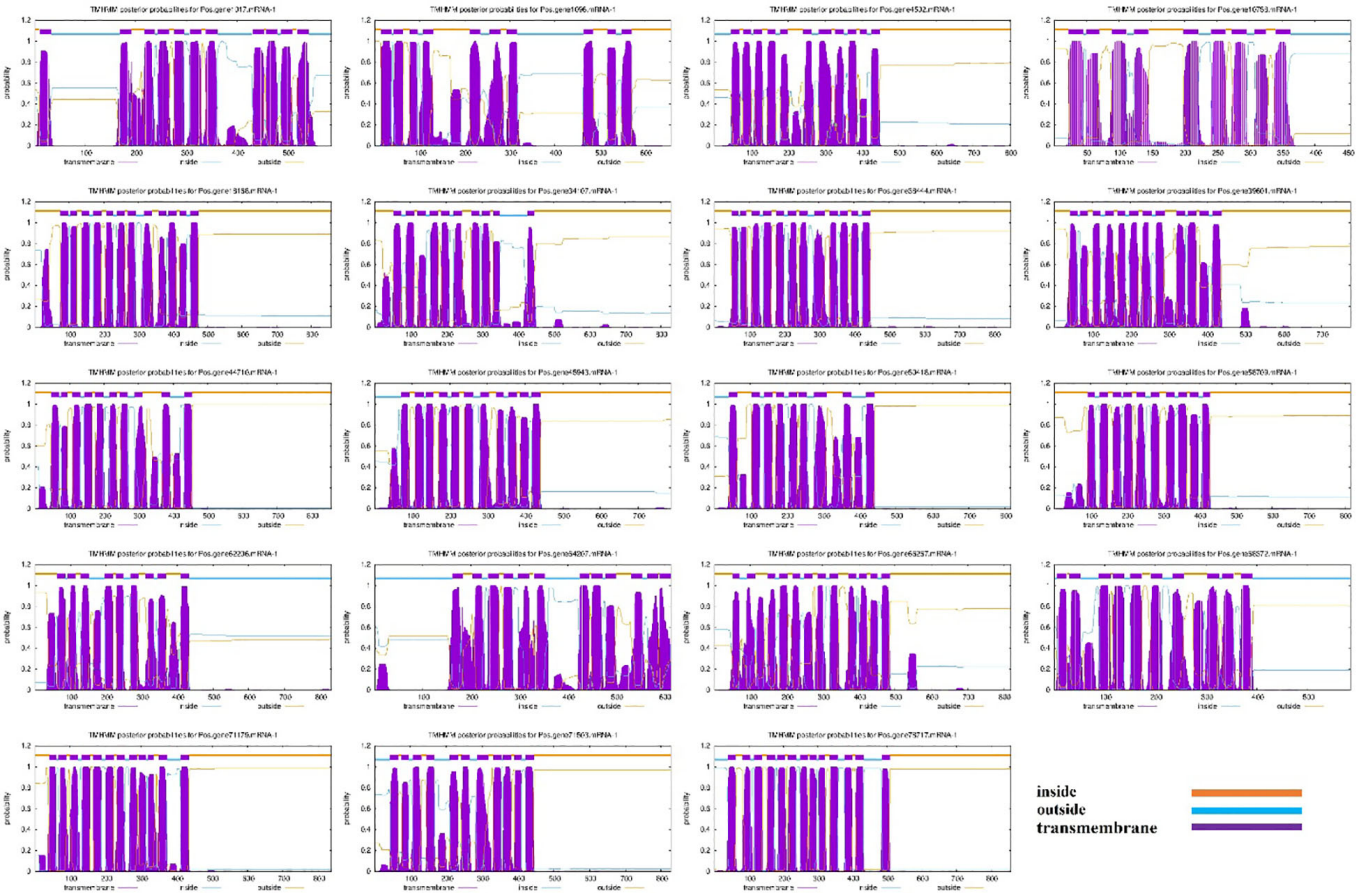
Transmembrane domains in PoSOS1 family members. The blue line denotes the outside, the orange line signifies the inside, and the purple line indicates the transmembrane region.

Nineteen (19) SOS1 transporter family genes were discovered in the *P. ostii* genome (**Supplementary Table S1**). The proposed protein sequences demonstrated significant diversity, with amino acid lengths ranging from 455 (Pos.gene10758.mRNA-1) to 859 (Pos.gene18168.mRNA-1). Seven genes produced polypeptides containing fewer than 800 amino acids, while the other 12 encoded polypeptides varied from approximately 803 to 859 amino acids, averaging a length of 762 amino acids. The statistical analysis indicated that the coding sequence (CDS) lengths varied from 1365 (*Pos.gene10758.mRNA-1*) to 2577 (*Pos.gene18168.mRNA-1*), with an average length of 2285. The relative molecular weight varied between 49803.54 and 95407.53 daltons (Da), with a mean of 83732.63 Da. The isoelectric points varied from 5.57 to 9.37, averaging 7.21. The protein Pos.gene62206.mRNA-1 exhibited the highest instability index (II) value of 46.64, whereas Pos.gene1017.mRNA-1 displayed the lowest instability index value of 31.68, resulting in a mean value of 38.11. The aliphatic index of the SOS1 family varied from 92.79 to 127.54, with a mean of 112.25. The grand average hydropathy index (GRAVY) values for 19 PoSOS1s varied from 0.263 to 0.724, signifying a hydrophobic nature. Identifying the subcellular location of SOS1 proteins will facilitate the comprehension of their molecular function. Predictions of subcellular localization suggested that members of the PoSOS1 family were predominantly located in the plasma membrane (**Table 1**). All PoSOS1 proteins were anticipated to possess transmembrane domains, with the number of domains ranging from 9 to 13 based on the specific gene. The PoSOS1 transporter family comprises three proteins with nine transmembrane domains and five proteins with ten transmembrane domains. Three proteins possessed 11 transmembrane domains, seven included 12, and Pos.gene78717.mRNA-1 exhibited a maximum of 13 transmembrane domains (**Figure 1**). Protein sequence alignment was conducted based on the presence of the Pfam (PF00999) domain and its similarity to query sequences (**Supplementary Figure S1**).

**Table 1:** General properties of PoSOS1 proteins.

Sequence I	AA^1^	CDS^2^	MW^3^	pI^4^	II^5^	AI^6^	GRAVY^7^	SL^8^
Pos.gene45943.mRNA-1	788	2364	86530.5	8.72	34.07	114.18	0.354	Plasma Membrane
Pos.gene4502.mRNA-1	803	2409	89838.3	9.37	43.65	117.2	0.382	Plasma Membrane
Pos.gene62206.mRNA-1	832	2496	92699.4	6.33	46.64	108.45	0.331	Plasma Membrane
Pos.gene66267.mRNA-1	820	2460	90806.8	7.65	35.64	112.06	0.424	Plasma Membrane
Pos.gene18168.mRNA-1	859	2577	93101.6	8.97	38.23	109.84	0.424	Plasma Membrane
Pos.gene39601.mRNA-1	777	2331	86459.1	8.48	41.71	118.03	0.464	Plasma Membrane
Pos.gene78717.mRNA-1	858	2574	95407.5	8.8	37.04	105.98	0.263	Plasma Membrane
Pos.gene64207.mRNA-1	614	1842	66912	5.86	32.1	126.82	0.724	Plasma Membrane
Pos.gene68372.mRNA-1	585	1755	61889.6	5.57	36.01	127.54	0.667	Plasma Membrane
Pos.gene71503.mRNA-1	828	2484	92680.9	8.94	41.13	114.14	0.285	Plasma Membrane
Pos.gene1096.mRNA-1	658	1974	73276.7	6.36	45.75	99.12	0.274	Plasma Membrane
Pos.gene34107.mRNA-1	829	2487	92023	8.87	35.78	108.72	0.361	Plasma Membrane
Pos.gene58709.mRNA-1	817	2451	89674	6.68	39.16	117.45	0.416	Plasma Membrane
Pos.gene71179.mRNA-1	837	2511	91335.6	6.02	37.51	111.15	0.367	Plasma Membrane
Pos.gene10758.mRNA-1	455	1365	49803.5	6.18	41.99	92.79	0.324	Plasma Membrane
Pos.gene1017.mRNA-1	587	1761	63466.6	5.77	31.68	124.53	0.679	Plasma Membrane
Pos.gene38444.mRNA-1	849	2547	93320.1	6.19	31.91	109.42	0.325	Plasma Membrane
Pos.gene50418.mRNA-1	816	2448	88223.9	9.24	39.5	109.79	0.392	Plasma Membrane
Pos.gene44710.mRNA-1	857	2571	93471	8.89	34.64	105.52	0.278	Plasma Membrane

AA^1^, Number of amino acids; CDS^2^, Coding Sequence; MW^3^, Molecular weight; pI^4^, Isoelectric point; II^5^, Instability Index; AI^6^, Aliphatic Index; GRAVY^7^, Grand average of hydropathicity; SL^8^, Subcellular Localization.

### 2.2 SOS1 protein phylogenetic relationships

**Figure 2:**
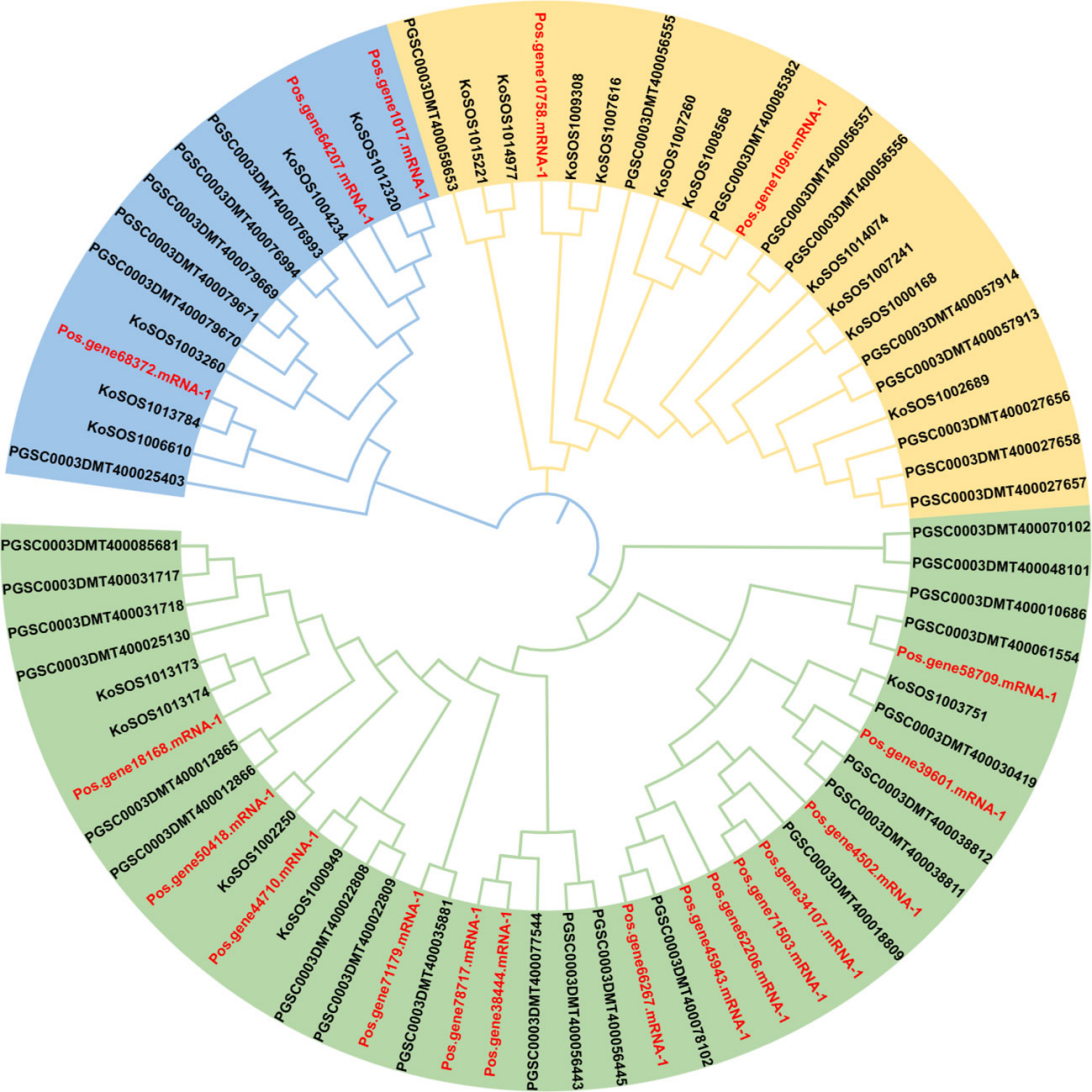
A phylogenetic analysis of SOS1 proteins from *Paeonia ostii* (Pos), *Kandelia obovata* (Ko), and Potato (PGSC) was conducted utilizing the maximum likelihood method. The SOS1 proteins were categorized into three distinct groups: Group 1 (light green), Group 2 (orange), and Group 3 (light blue), with each group denoted by a unique color. The red color text showed *P. ostii* SOS1 proteins.

The evolutionary relationships between several plant species were evaluated using a phylogenetic tree. *Paeonia ostii, Kandelia obovata*, and potato full-length protein sequences were obtained. A neighbor-joining tree was created utilizing 76 SOS1 proteins by repeated sequence alignment with the MEGA-X MUSCLE tool (**Figure 2**). This phylogenetic tree utilized protein sequences from 19 (*Paeonia ostii*), 20 (*Kandelia obovata*), and 37 (*Solanum tuberosum*). The SOS1 proteins were categorized into three groups: group 1 (light green), group 2 (orange), and group 3 (light blue) (**Figure 2**). The group had 40 SOS1 proteins, which were classified into various categories according to their species-specific traits. This subgroup consisted of 14 *Paeonia ostii* (Pos) proteins, five *Kandelia obovata* (Ko) proteins, and 21 Potato (PGSC) proteins. Group II comprised 22 SOS1 proteins, including two from *Paeonia ostii* (Pos), ten from *Kandelia obovata* (Ko), and ten from Potato (PGSC). Group III comprised 14 SOS1 proteins, including three from *Paeonia ostii* (Pos), five from *Kandelia obovata* (Ko), and six from Potato (PGSC). The data presented in **Figure 2** indicate that Group I displayed a higher abundance of PoSOS1 members than Groups II and III. The PoSOS1 proteins are more similar to their counterparts in *Kandelia obovata* than those in the Potato.

### 2.3 Locations of *PoSOS1s* on chromosomes in the *Paeonia ostii* genome

**Figure 3:**
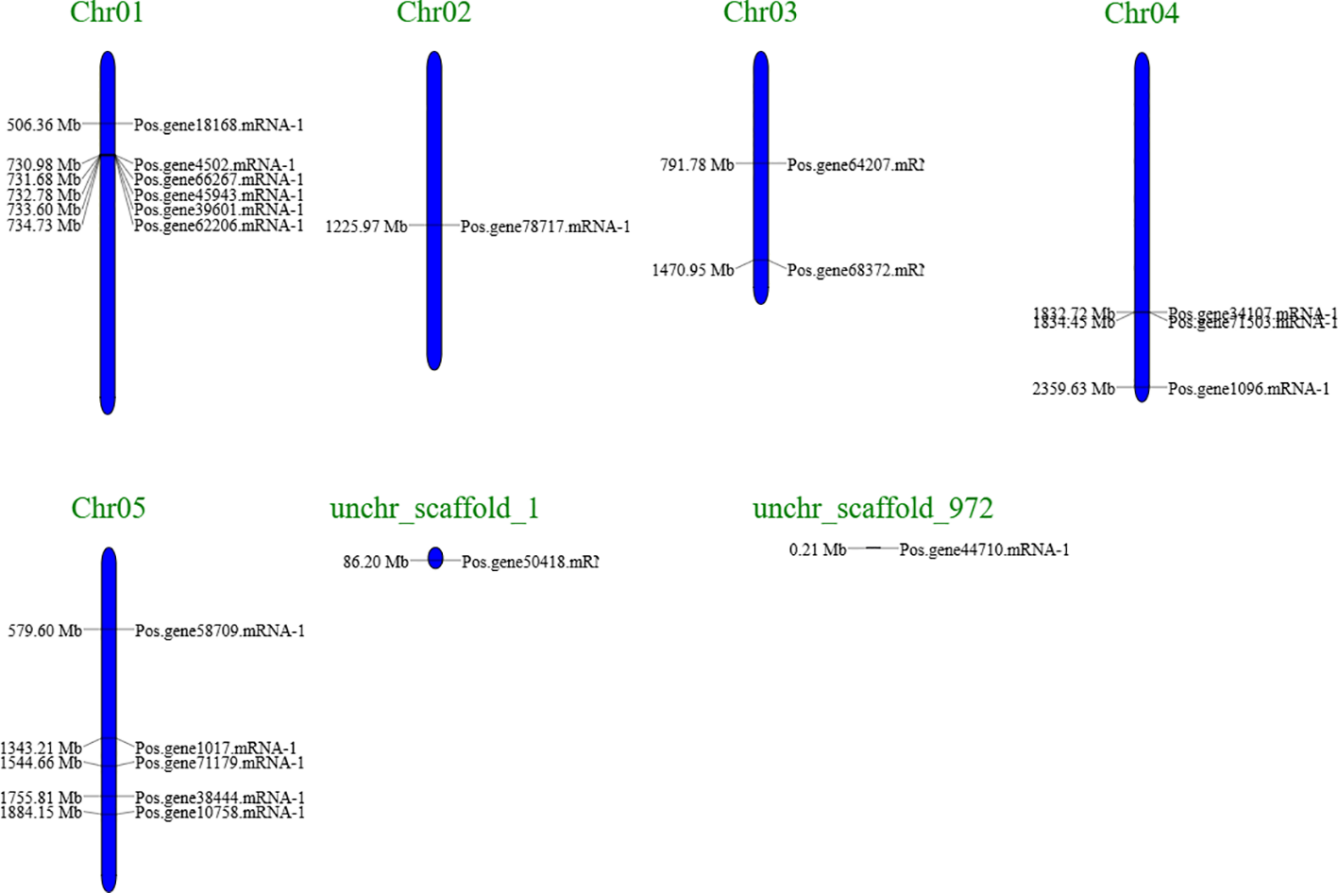
A schematic representation of the *SOS1* gene distribution in *Paeonia ostii* included two scaffolds and five chromosomes, with the gene’s name on the right. The black inscriptions on the chromosomes denote the position of the *SOS1* genes. The chromosomal numbers are located at the apex of each chromosome (Chr).

A chromosomal map of PoSOS1s was created using the genome sequences of *Paeonia ostii* to understand better the process underlying the genomic distribution of PoSOS1s on the species’ chromosomes (**Figure 3**). The map analysis indicated that 17 of the 19 PoSOS1 genes resided on the *P. ostii* chromosome, while the remaining two were on the scaffold. Genes that encode members of the SOS1 protein family were located on all five chromosomes. Six genes (*Pos.gene18168.mRNA-1, Pos.gene39601.mRNA-1, Pos.gene4502.mRNA-1, Pos.gene45943.mRNA-1, Pos.gene62206.mRNA-1, Pos.gene66267.mRNA-1*) were located on chromosome 1, one gene (*Pos.gene78717.mRNA-1*) on chromosome 2, two genes (*Pos.gene64207.mRNA-1, Pos.gene68372.mRNA-1*) on chromosome 3, three genes (*Pos.gene1096.mRNA-1, Pos.gene34107.mRNA-1, Pos.gene71503.mRNA-1*) on chromosome 4, and five genes (*Pos.gene1017.mRNA-1, Pos.gene10758.mRNA-1, Pos.gene38444.mRNA-1, Pos.gene58709.mRNA-1, Pos.gene71179.mRNA-1*) on chromosome 5. While the two genes *Pos.gene50418.mRNA-1* and *Pos.gene44710*.*mRNA-1* were located on unchr_scaffold_1 and unchr_scaffold_972. The genes were predominantly uniformly allocated throughout the five chromosomes and two scaffolds (**Figure 3**; **Supplementary Table S3**).

### 2.4 PoSOS1s collinearity analysis

**Figure 4:**
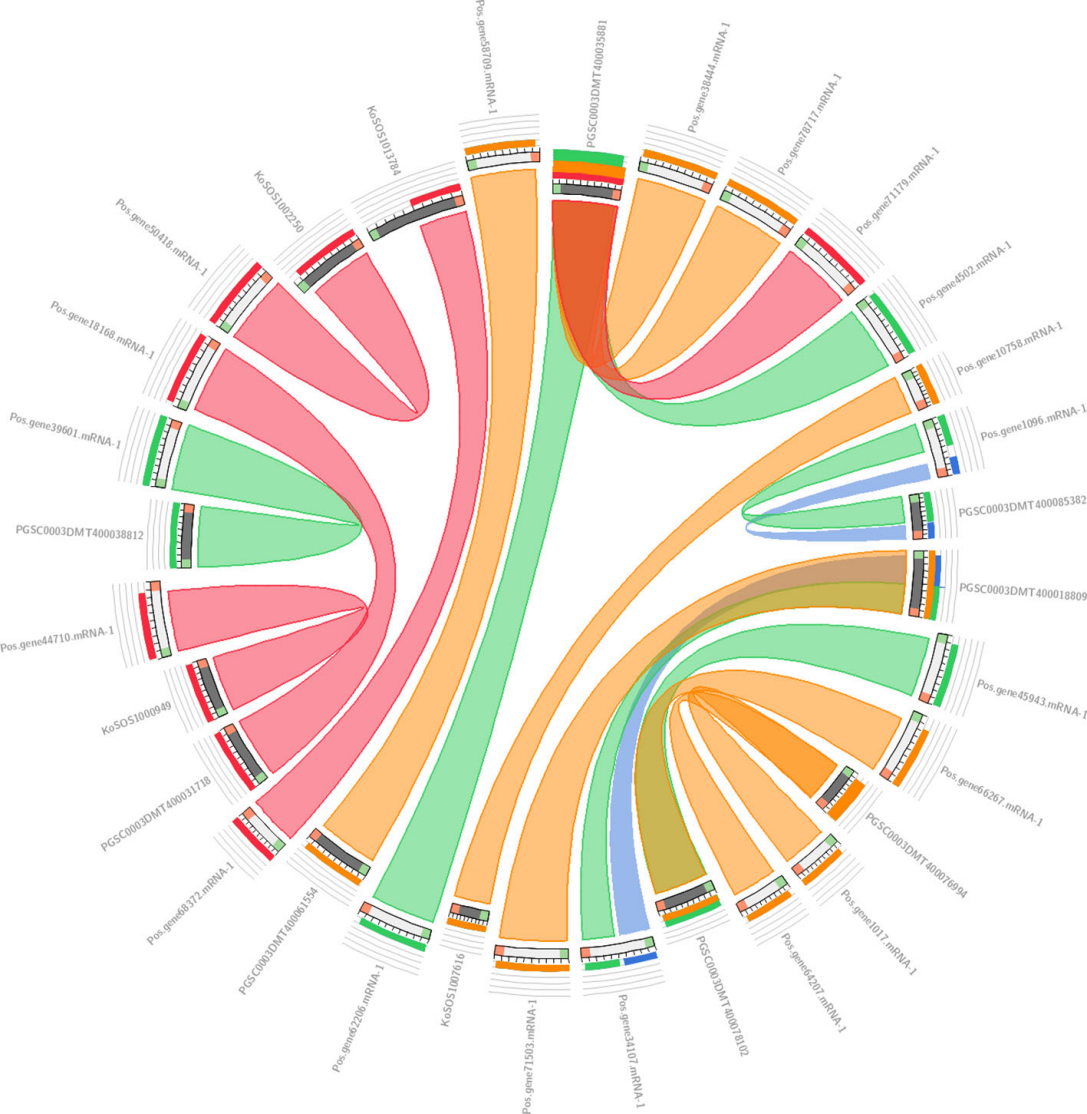
Collinearity analysis results between *Paeonia ostii*, *Kandelia obovata*, and Potato. In Circos, the colors blue <=25%, green <=50%, orange <=75%, and red >75% all display their sequential identity in the explosion.

The collinearity analysis results are presented in **Figure 4**, which shows the genomic relationships between *Paeonia ostii*, *Kandelia obovata*, and Potato. In this analysis, we used 19 *Paeonia ostii* genes as a query to search against a database of 57 genes, comprising 20 genes from *Kandelia obovata* and 37 genes from Potato. The results were visualized using a ‘score/max’ ratio coloring scheme, where blue (≤0.25) indicates low collinearity, green (≤0.50) indicates moderate collinearity, orange (≤0.75) indicates high collinearity, and red (>0.75) indicates very high collinearity. As shown in **Figure 4**, a clear pattern of collinearity is evident between the *Paeonia ostii* genes and their counterparts in *Kandelia obovata* and Potato, with several regions exhibiting high collinearity (orange to red). These findings suggest that conserved genomic areas exist across the three species.

### 2.5 Investigation of the *SOS1* gene structure and conserved motifs

**Figure 5:**
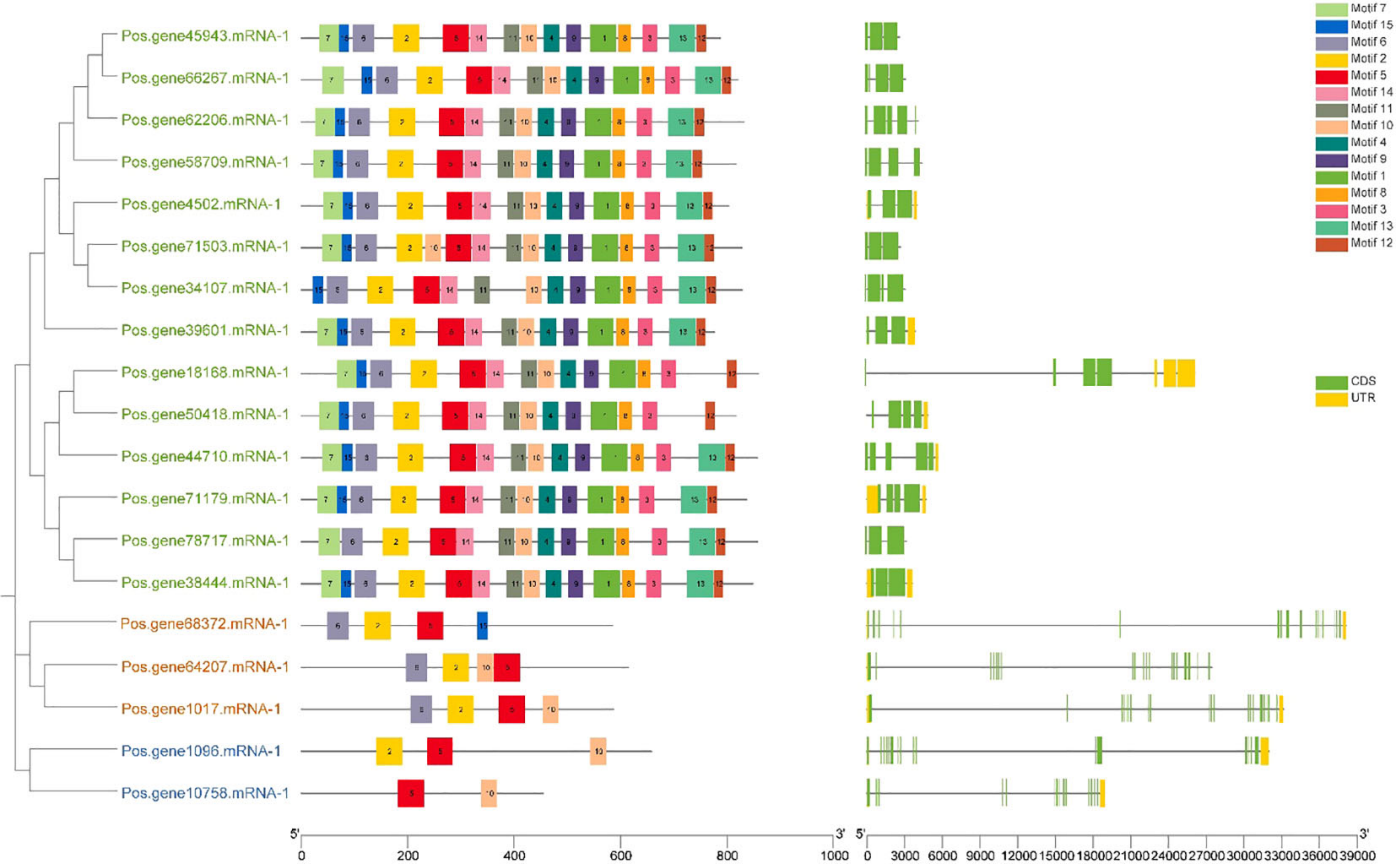
Investigations were undertaken about the gene structure and motif composition of the SOS1 gene family in *Paeonia ostii*. In the phylogenetic tree, the green text shows Group I, the orange color shows Group II and the blue text shows Group III. The *SOS1* genes identified in the genome were categorized into three distinct categories based on their evolutionary relationships, with particular attention to the gene structure of the *SOS1s*. The UTR sections are graphically represented in yellow, whereas the CDS or exons are also depicted in green. A black horizontal line denotes introns. Furthermore, the conserved patterns in the SOS1s are defined by a specific letter. Vibrantly hued boxes featuring various patterns are exhibited.

An investigation of gene structure and conserved motifs was done to elucidate the association between the structure and function of PoSOS1 proteins, creating distinct phylogenies. A phylogenetic tree was created using the individual sequences of the SOS1 protein. The SOS1 proteins were classified into three groups: Group 1, green color text; Group II, orange color text; and Group III, blue color text. The study examined the exon-intron configurations of the *SOS1* genes to explore gene expansion within the *Paeonia ostii* family. Examining exon-intron architectures and conserved motifs, as depicted in **Figure 5**, we identified that the *SOS1* gene displays a variable number of exons (from 3 to 20) and introns (from 4 to 21). Group I included a relatively limited number of introns, ranging from 4 to 9, whereas Groups II and III exhibited a more extensive range, from 19 to 21. *Pos.gene64207.mRNA-1* exhibited the maximum quantity of exons (20) and introns (21), as illustrated in **Figure 5**. The study demonstrated that the *SOS1* genes in *Paeonia ostii* exhibit a highly conserved gene structure, signifying a notable similarity to those in closely related species. To further examine the evolutionary variety of the PoSOS1 family, the conserved motifs of the 19 PoSOS1 proteins were analyzed using MEME online software, identifying 15 distinct conserved motifs (designated motifs 1–15) (**Figure 5**). The conserved motifs in all *SOS1* genes displayed a variation of two to 15. The results showed that motifs 2, 5, and 6 have been detected in 17 proteins. Motif 7 was identified in 13 proteins, and motifs 1, 3, 4, 8, 9, and 10 were identified in 14 proteins. We also found that 15 motifs were primarily distributed in Groups 1 (14-15), while four motifs, including 2, 5, 6, 10, and 15 were found in Group 2, and motifs 2, 5, and 10 were found in Group 3. Motif 10 identified two copies in protein Pos.gene71503.mRNA-1.

### 2.6 Variation across the SOS1 family in terms of 3D and secondary structure

**Figure 6:**
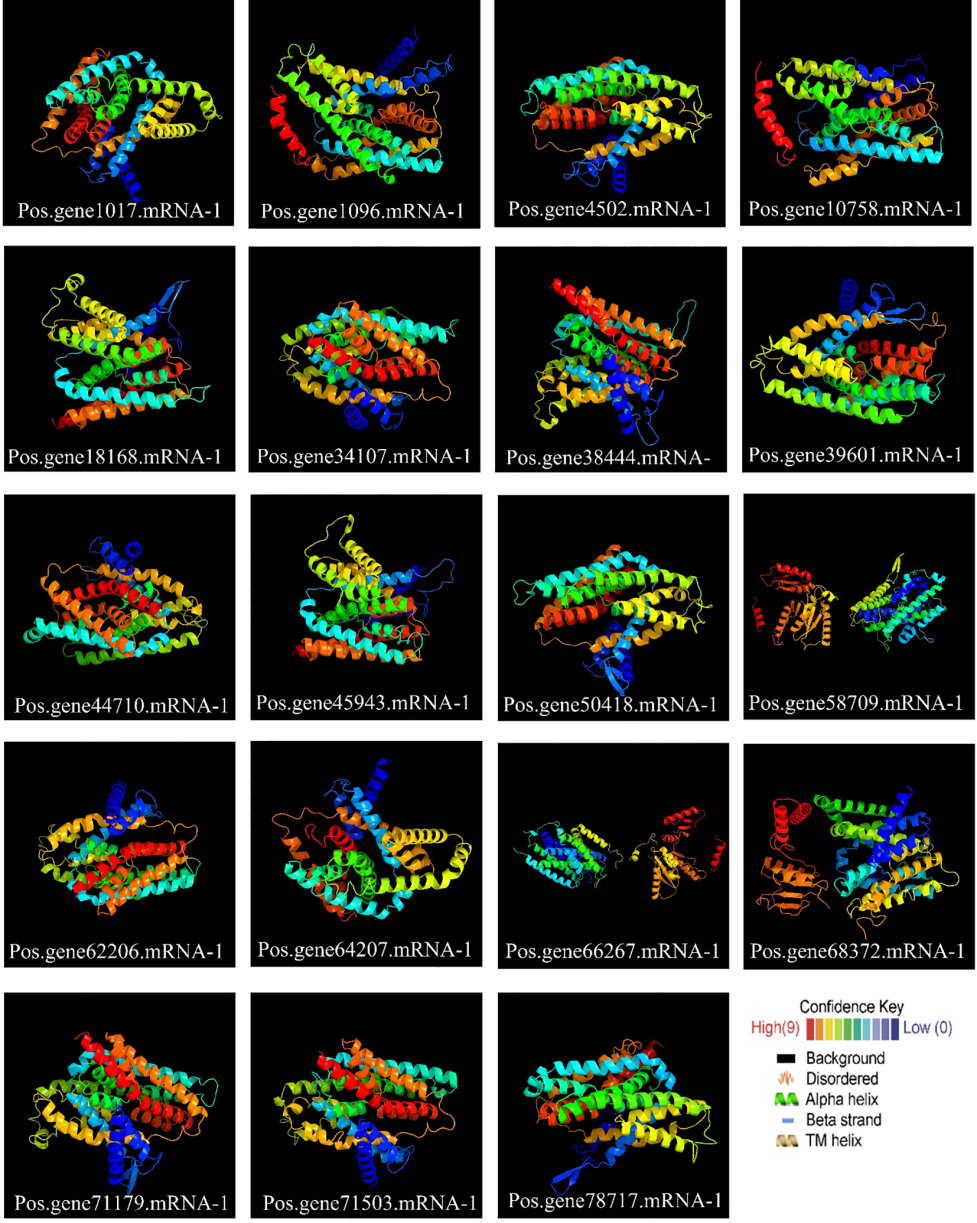
Protein 3D structures and modeling. Also, see **Supplementary Table S4** for detailed results.

All PoSOS1 proteins were subjected to 3D modeling (**Figure 6**), and the detailed results of the projected templates are presented in **Supplementary Table S2**. Four unique templates were discovered, including c5bz2A, c8pvrA, c4bwzA, and c5bz3A, which displayed a 100% confidence level. The high confidence level of various templates proposes reliable structural estimates. Briefly, six proteins, including Pos.gene34107.mRNA-1, Pos.gene62206.mRNA-1, Pos.gene64207.mRNA-1, Pos.gene66267.mRNA-1, Pos.gene71179.mRNA-1, and Pos.gene71503.mRNA-1 were modeled based on the “c4bwzA” template, while five proteins, including Pos.gene1017.mRNA-1, Pos.gene18168.mRNA-1, Pos.gene38444.mRNA-1, Pos.gene45943.mRNA-1, and Pos.gene68372.mRNA-1 were modeled based on the “c5bz2A” template. The other six proteins include Pos.gene39601.mRNA-1, Pos.gene44710.mRNA-1, Pos.gene4502.mRNA-1, Pos.gene50418.mRNA-1, Pos.gene58709.mRNA-1, and Pos.gene78717.mRNA-1 were modeled based on the “c5bz3A” template, while two proteins, including Pos.gene10758.mRNA-1 and Pos.gene1096.mRNA-1 were modeled based on the “c8pvrA” template. The flexible structures emphasized by coils may aid the functional adaptability of PoSOS1 proteins.

The secondary protein structure results showed transmembrane (TM) helices, beta strands, alpha helices, and protein disorder (**Supplementary Table S4**). Among all the 19 PoSOS1 proteins, the alpha helix ranged from 43% (Pos.gene78717.mRNA-1) to 65% (Pos.gene64207.mRNA-1), beta-strand ranged from 0% (Pos.gene10758.mRNA-1) to 11% (Pos.gene34107.mRNA-1), transmembrane (TM) helix ranged from 28% (Pos.gene78717.mRNA-1) to 45% (Pos.gene10758.mRNA-1 and Pos.gene64207.mRNA-1), and disordered ranged from 12% (Pos.gene68372.mRNA-1) to 24% (Pos.gene1017.mRNA-1) (**Supplementary Table S4**). These scores advise significant adaptability in the structural elements of PoSOS1 proteins, implying diverse functional roles. The broad scope of secondary structure elements highlights these proteins’ structural complexity and possible adaptability.

### 2.7 Prediction of cis-elements in the promoter sequences of *PoSOS1* genes

**Figure 7:**
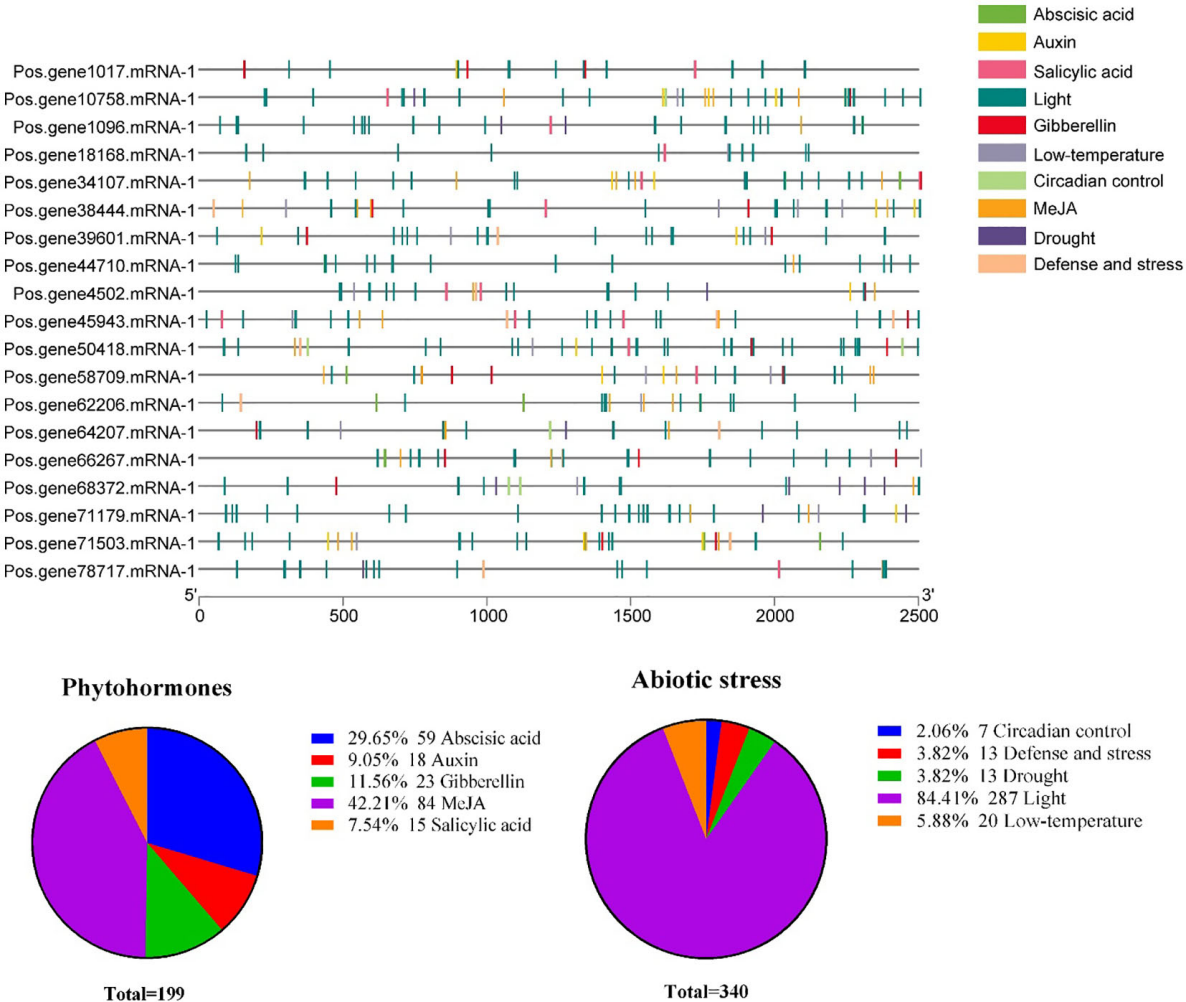
The anticipated cis-regulatory regions within the promoter of the *PoSOS1* genes. This legend employs diverse colors to represent distinct cis-elements metaphorically. Diverse cis-elements involved in abiotic stress and phytohormone responses are shown with diverse colors.

**Figure 8:**
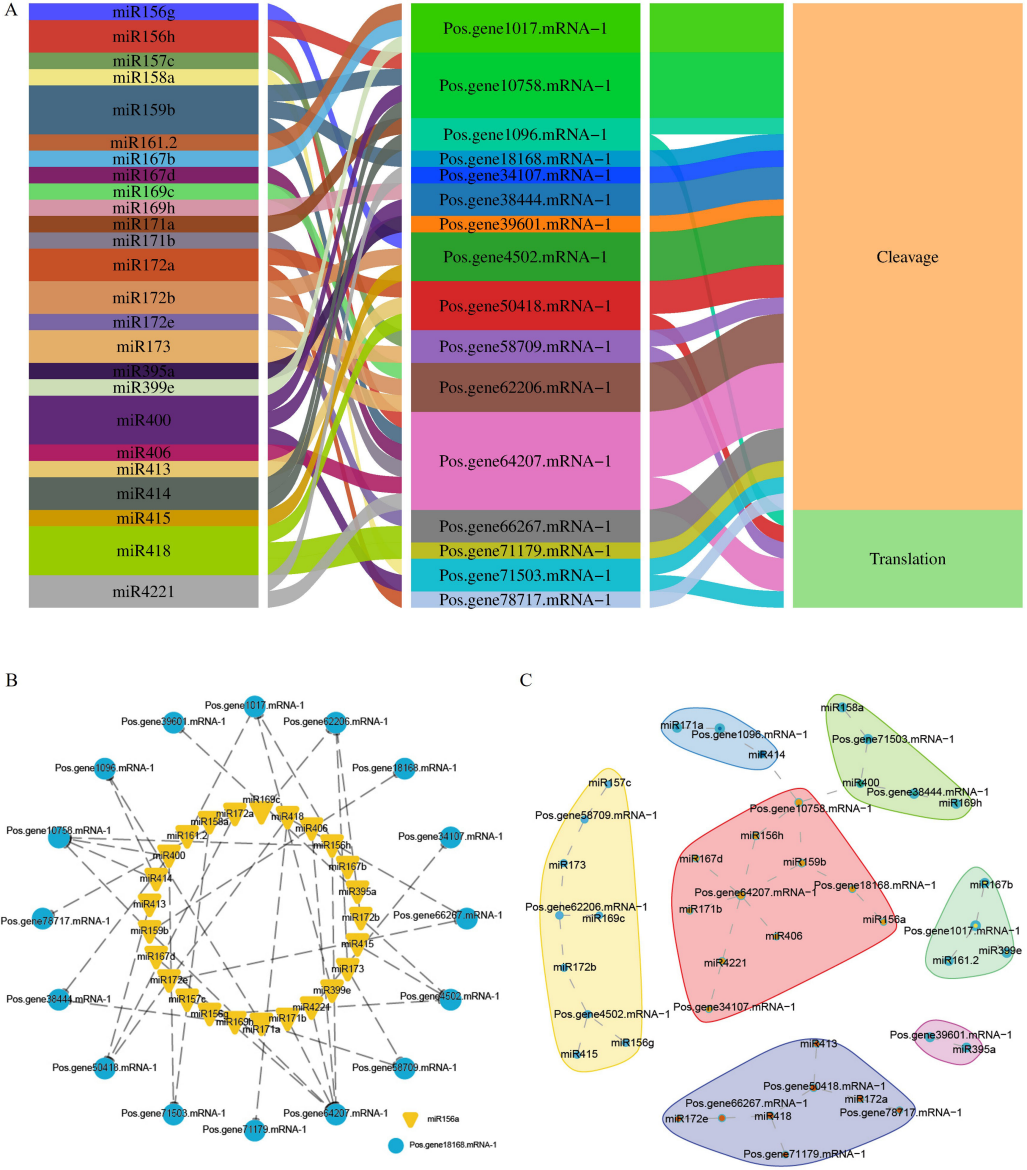
miRNA interactions with *PoSOS1* genes. **(A)** A Sankey diagram shows the connections among miRNAs, target genes, and their inhibitory effects. **(B, C)** Correlation and network analysis display the predicted miRNA interactions with *PoSOS1* genes.

To clarify which hormonal, environmental stress, or developmental-related signal elements are involved in these *PoSOS1s*, we performed a promoter analysis using the PlantCARE server. A total of 10 critical cis-acting elements, including abscisic acid, auxin, circadian control, defense and stress, drought, gibberellin, light, low-temperature, MeJA, and salicylic acid, were annotated from the 2,500 bp upstream promoter region of the 19 *PoSOS1* genes (**Figure 7**). Concerning the *PoSOS1* genes, it is noteworthy that among all 10 cis-acting elements, light-responsive cis-regulatory elements accounted for the most significant proportion (84.41%). Hormone-related cis-elements, including abscisic acid, auxin, gibberellin, MeJA, and salicylic acid, accounted for 29.65%, 9.05%, 11.56%, 42.21%, and 7.54%. In contrast, abiotic stress-related elements, including circadian control, defense and stress, drought, light, and low-temperature, accounted for 2.06%, 3.82%, 3.82%, 84.41%, and 5.88% of the total (**Figure 8**). The variation in the response components demonstrated the regulatory functions of *PoSOS1* genes in numerous physiological and biological processes (**Supplementary Table S5**).

### 2.8 Ka/Ks calculation and Protein pairwise similarity matrix of PoSOS1 proteins

The selection pressure between tandem and segmental duplications was evaluated by computation of the Ka/Ks ratio. Concerning the evolutionary mechanism, genes are subjected to various forms of selection pressure, indicated by the ratios of Ka/Ks < 1, Ka/Ks = 1, and Ka/Ks > 1, corresponding to purifying selection, neutral selection, and positive selection. A collinearity association test investigated gene duplication within the PoSOS1 gene family. For five pairs of PoSOS1 members, the Ka/Ks ratio was <1, suggesting that all PoSOS1 duplicated genes were under purifying or negative selection. In one pair, Pos.gene18168.mRNA-1 and Pos.gene50418.mRNA-1, the Ka (nonsynonymous) value was 0.2268, but the Ks (synonymous) value was NaN, so the Ka/Ks value was NaN (**Table 2**). To investigate the similarities among the 19 PoSOS1 proteins, we generated a protein pairwise similarity matrix using TBtool v2.516. The matrix shows the pairwise similarity scores between each pair of proteins, ranging from 2.57 to 73.71. The similarity matrix is shown in **Supplementary Table S6**. The matrix is symmetric, with the diagonal elements representing the self-similarity of each protein.

**Table 2:** Ka/Ks analysis of *PoSOS1* genes.

Gene 1	Gene 2	Ka	Ks	Ka/Ks
*Pos.gene45943.mRNA-1*	*Pos.gene66267.mRNA-1*	0.3655	1.32724	0.27538529
*Pos.gene71503.mRNA-1*	*Pos.gene34107.mRNA-1*	0.30428	0.7975	0.38153951
*Pos.gene18168.mRNA-1*	*Pos.gene50418.mRNA-1*	0.2268	NaN	NaN
*Pos.gene78717.mRNA-1*	*Pos.gene38444.mRNA-1*	0.32426	0.90122	0.3598057
*Pos.gene64207.mRNA-1*	*Pos.gene1017.mRNA-1*	0.11317	1.13948	0.09931784
*Pos.gene1096.mRNA-1*	*Pos.gene10758.mRNA-1*	0.7239	2.53044	0.28607878

### 2.9 Identification of miRNAs that target *PoSOS1* genes

This study identified 38 miRNAs that target 19 genes from 18 distinct families (**Figure 8A**). In total, 27 miRNAs influenced 13 PoSOS1 genes through cleavage, while six miRNAs affected five PoSOS1 genes via translation. Notably, four miRNAs—miR159b, miR400, miR414, and miR4221—demonstrated inhibitory effects on different genes (**Figure 8A**). These findings indicate that various miRNAs play a role in the post-transcriptional regulation of PoSOS1 genes by interacting with them through cleavage and translation. **Figures 8B, C** illustrates the correlations and networks between miRNA-targeted and PoSOS1 genes. The results indicated that the Pos.gene1017.mRNA-1 was targeted by three miRNAs (miR161.2, miR167b, miR399e), whereas Pos.gene10758.mRNA-1 was targeted by four miRNAs (miR156h, miR159b, miR400, miR414). Six miRNAs, such as miR156h, miR167d, miR171b, miR406, miR159b, and miR4221 targeted one gene (*Pos.gene64207.mRNA-1)*. Similarly, the six genes, including *Pos.gene1096.mRNA-1*, *Pos.gene18168.mRNA-1*, *Pos.gene38444.mRNA-1*, *Pos.gene58709.mRNA-1*, *Pos.gene66267.mRNA-1*, and *Pos.gene71503.mRNA-1* were targeted by each two miRNAs (**Figures 8B, C**).

### 2.10 Expression analysis of PoSOS1 genes under salt stress

**Figure 9:**
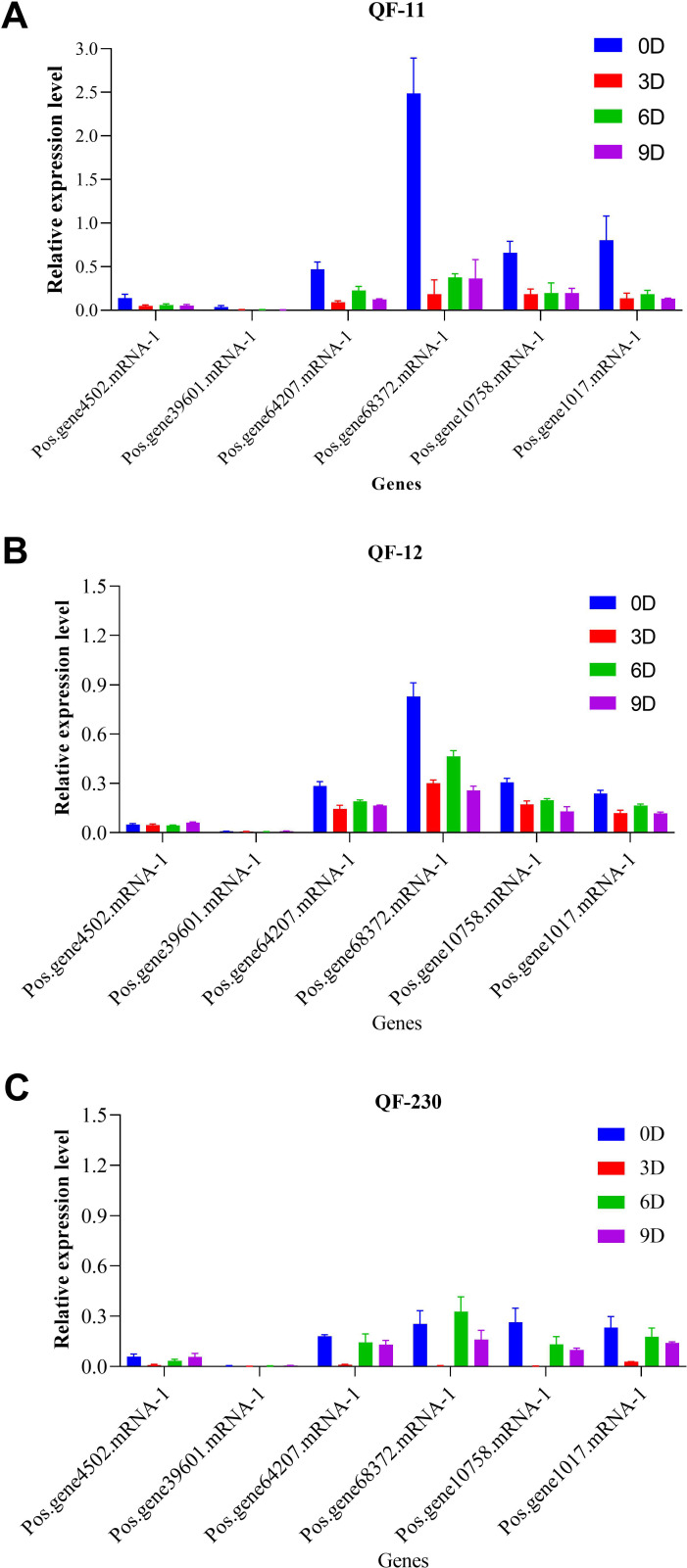
Expression levels of the *SOS1* genes in the three cultivars QF-11, QF-12, and QF-230 leaves of seedling-stage *P. ostii* plants under various salt stress conditions, as assessed by qRT-PCR analysis. The vertical axis represents relative gene expression, whereas the horizontal axis denotes genes.

Quantitative RT-PCR analysis of six *SOS1* genes in salt-stressed *Paeonia suffruticosa* cultivars (QF-11, QF-12, and QF-230) revealed distinct temporal expression patterns. Five genes showed significant downregulation across all time points compared to controls; their expression was markedly higher on day 6 than on days 3 and 9, with day 3 exhibiting the lowest transcript levels (**Figure 9**). Notably, *Pos.gene68372.mRNA-1* displayed unique dynamics, peaking in upregulation on day 6 before declining sharply by day 9 (**Figure 9**). Cultivar-specific responses were evident, with QF-11 showing the most severe suppression on day 3. At the same time, all cultivars shared the trend of partial transcriptional recovery on day 6, followed by renewed suppression on day 9, suggesting a biphasic stress response on day 6, representing a critical transitional phase before sustained repression.

## 3 Discussion

Salt stress is a major abiotic stress that severely impacts plant growth, development, and productivity worldwide (Hussain et al., 2021). *Paeonia ostii*, an economically significant ornamental and traditional medicinal plant in China, is no exception (Yang et al., 2025). Elucidating the underlying molecular mechanisms of salt stress tolerance in *P. ostii* is crucial for developing strategies to improve its salt tolerance and ensure sustainable cultivation (Li et al., 2024; Chen et al., 2024). The Salt Overly Sensitive 1 (*SOS1*) gene family plays a vital role in plant salt tolerance by regulating ion homeostasis and transport (Shang et al., 2024). Our results revealed 19 *SOS1* genes in the *P. ostii* genome, exhibiting distinct structural and functional characteristics. Multiple *SOS1* genes in *P. ostii* suggest that this plant species may have evolved complex mechanisms to regulate ion homeostasis and transport under salt stress conditions (Zhu, 2003). Researchers identified 119, 37, 20, and 12 SOS1 gene families in wheat (Jiang et al., 2021), potato (Liang et al., 2023), *Kandelia obovata* (Shang et al., 2024), and tuber mustard (Cheng et al., 2019), respectively. The SOS1 protein, encoded by the *SOS1* gene, functions as a proposed plasma membrane Na^+^/H^+^ antiporter, facilitating the expulsion of Na^+^ ions from plant cells. As a result, it keeps the K^+^ and Na^+^ levels in plant cells balanced and stops the accumulation of Na^+^ in plant cells (Zhang et al., 2022). Our research identified more *SOS1* gene families within the *P. ostii* genome, which is significant. Our research reveals the presence of 19 *SOS1* homologs in the genome of *P. ostii*. The quantity of *SOS1* genes in the *P. ostii* genome surpasses that in tuber mustard, primarily because *P. ostii* is a diploid species (Cheng et al., 2019).

The chromosomal localization of the *SOS1* genes on five chromosomes and two scaffolds indicates that these genes may have undergone duplication and rearrangement events during the evolution of *P. ostii*. Comprehending *PoSOS1’s* subcellular localization is essential for elucidating its function. All KoSOS1 proteins have been demonstrated to localize to the plasma membrane in *Paeonia ostii*, corroborating findings from prior work in Potato (Liang et al., 2023). Phylogenetic analysis clustered the PoSOS1 genes into three subfamilies, suggesting that these genes may have distinct functions and regulatory mechanisms. Interestingly, all three plant species had three subfamilies, indicating that genetic expansion occurred before these species diverged. A phylogenetic analysis showed that the closest link was between *PoSOS1* and wheat (*TaSOS1*) (Jiang et al., 2021).

One possible source of information on functional diversification during evolution is the intron-exon gene structure (Hussain et al., 2022). The gene structure of *PoSOS1* was analyzed, leading to the identification of three different patterns. The *PoSOS1* genes exhibited exon counts between 3 and 20, whereas intron counts varied from 4 to 21. The gene structure pattern seen in this study corresponds with the gene structure patterns identified in potatoes, as documented in prior research (Shang et al., 2024). The cis-elements and functional characterization of *SOS1* gene promoters have been identified in many species, including *Kandelia obovate* (Shang et al., 2024), wheat (Jiang et al., 2021), tuber mustard (Cheng et al., 2019), and potato (Liang et al., 2023). This work examined the promoter cis-elements of *SOS1* homologs in *P. ostii*. All promoters exhibited a variety of cis-elements responsive to plant hormones and abiotic stressors. The promoter cis-elements of *SOS1* genes in *P. ostii*, as indicated by prior studies in other species, suggest that the expression patterns of *SOS1* homologs are modulated by hormonal and abiotic stresses, implying that these homologs may be involved in mediating the response of *P. ostii* to such stresses. The results indicated that *PoSOS1s* may significantly contribute to the response to phytohormones and abiotic stressors (Shang et al., 2024; Liang et al., 2023; Cheng et al., 2019).

Gene expression analysis uncovered six differentially expressed *SOS1* genes under salt stress, with only one gene up-regulated in the QF230 cultivar and five genes down-regulated compared to the control. The up-regulation of the *SOS1* gene under salt stress suggests that these genes may be crucial in maintaining ion homeostasis and transport in *P. ostii*. Similar findings were made regarding the up-and-down-regulation of *SOS1* genes in potatoes (Liang et al., 2023) and *Kandelia obovata* (Shang et al., 2024) during salt stress. Employing the *PoSOS1* genes as practical genetic modifiers would increase plants’ and crops’ resistance to elevated salt concentrations.

## 4 Materials and methods

### 4.1 Identification and characterization of *SOS1* genes in *Paeonia ostii* genome

The genomic data of *P. ostii* (CNA0050666) were obtained from the China National Gene Bank (https://ftp.cngb.org/pub/CNSA/data5/CNP0003098/CNS0560369/CNA0050666/) (Yuan et al., 2022). The screening aimed to identify all members of the SOS1 gene family in *P. ostii*. The hidden Markov model (HMM) files for the SOS1 domain (PF00999) were obtained from the Pfam database (http://pfam-legacy.xfam.org/, accessed on 17 August 2023). The HMMER tool was subsequently employed to discover the SOS1 proteins inside the *P. ostii* genome. The SOS1 (Na^+^/H^+^ exchanger) domain of all candidate SOS1 proteins was identified using the conserved domain database Batch CD-Search (https://www.ncbi.nlm.nih.gov/Structure/bwrpsb/bwrpsb.cgi). Nineteen (19) potential *SOS1* genes were found. The online platform (http://web.expasy.org/Compute-pI/) was employed to forecast fundamental physicochemical characteristics. TMHMM-2.0: https://services.healthtech.dtu.dk/service.php?TMHMM-2.0 was utilized to predict transmembrane helices in proteins.

### 4.2 Phylogenetic tree construction

Using MEGA12’s default parameters, MUSCLE was used to align several sequences. Phylogenetic analysis was conducted using SOS1 protein sequences from three species: *Paeonia ostii*, *Kandelia obovata*, and *Solanum tuberosum*. The neighbor-joining (NJ) method, utilizing 1000 bootstrap replicates, was employed to generate a phylogenetic tree. A pairwise deletion mode was employed to validate the dissimilarity regions capable of producing the topology of the NJ tree. The phylogenetic tree was visualized and refined using iTOL (https://itol.embl.de/) (Hussain et al., 2023).

### 4.3 Prediction of chromosomal localization

We utilized the China National Gene Bank (https://ftp.cngb.org/pub/CNSA/data5/CNP0003098/CNS0560369/CNA0050666/, to ascertain the genomic locations and protein sequences of all *Paeonia ostii SOS1* genes, and we evaluated the distribution of *SOS1* genes on scaffolds or chromosomes (Yuan et al., 2022). *SOS1* genes were identified on the chromosomes of *Paeonia ostii* utilizing MapGene2Chromosome (MG2C; http://mg2c.iask.in/mg2c v2.0/).

### 4.4 Collinearity analysis and Ka/Ks calculation

The syntenic relationships of the *SOS1* genes among *Paeonia ostii*, *Kandelia obovata*, and Potato were analyzed using the Circletto online program (https://bat.infspire.org/circoletto/). *Paeonia ostia* (Pos) protein sequences were utilized as query sequences, whereas *Kandelia obovata* (Ko) and 21 Potato (PGSC) protein sequences served as databases. Distinct colors—blue, green, orange, and red—represented <25%, <50%, <75%, and >75% identity of the protein sequences in the BLAST analysis, respectively. Ultimately, Circos was computed and illustrated according to the blast score and subsequently updated in Adobe Illustrator CC2019. The Ka and Ks substitution rates and the Ka/Ks ratios of nucleotides were annotated and computed using TBtool v2.516 (Waheed et al., 2024).

### 4.5 Analysis of conserved motif and gene structure

The gene structure of *PoSOS1s* was examined using TBtool v2.516 software, utilizing the gene structure annotation file in GFF3 format from *P. ostii*. The conserved motifs of PoSOS1s were examined using MEME (Multiple Em for Motif Elicitation) v5.3.3 (http://meme-suite.org/tools/meme) with default parameters. The XML file containing motif pattern data acquired from MEME was utilized to produce schematic diagrams of motif distribution using TBtool v2.516 software (Waheed et al., 2024).

### 4.6 Three-dimensional structure and subcellular localization

Secondary structures and 3D modeling of all SOS1 proteins were performed via the Phyre2 online site with default settings (https://www.sbg.bio.ic.ac.uk/phyre2/html/page.cgi?id=index).

The subcellular localization of the SOS1 family genes was predicted using the online program CELLO v.2.5, a software application for subcellular localization prediction accessible at http://cello.life.nctu.edu.tw/.

### 4.7 Analysis of cis-regulatory elements

SOS1 family members’ upstream sequences totaling 2,500 bp were gathered from the *Paeonia ostii* genome assembly database. The PlantCARE tool (http://bioinformatics.psb.ugent.be/webtools/plantcare/html/) identified CREs among the acquired sequences. Based on the frequency count of each CRE motif, the most common CREs found for the *SOS1* genes were used to create **Figure 6** in TBtool v2.516.

### 4.8 Estimation of miRNA targets and their functional annotation

The psRNATarget website was utilized to identify potential miRNA target sites. The CDS sequences of all *PoSOS1* genes were uploaded to the site (https://www.zhaolab.org/psRNATarget/), using Arabidopsis data as a reference under the default settings parameters. The diagram depicting the interaction between miRNA and target genes and *PoSOS1* genes was generated using https://www.bioinformatics.com.cn/plot_basic_miRNA_target_network_plot_197.

### 4.9 Plant material and environmental conditions

Three 6-year-old *Paeonia suffruticosa* (tree peony) individuals (cultivar codes: QF-11, QF-12, QF-230) cultivated at the *Paeonia* Germplasm Repository of Wuhan Academy of Agricultural Sciences Forestry and Fruit Research Institute (Hongshan District, Wuhan, China) were selected for salt stress experiments. At the initial bud stage, tender leaf samples were collected from three untreated control plants at 10:30 AM. Subsequently, each plant was irrigated with 4 L of 10 g/L sodium chloride (NaCl) solution. The rhizosphere soil was immediately covered with a waterproof film to prevent rainwater infiltration. Treated leaves were sampled at 10:30 AM on days 3, 6, and 9 post-treatments. All collected tissues were flash-frozen in dry ice and stored at -80°C for subsequent RNA extraction. Six SOS1 gene family members were identified from the published *Paeonia ostii* ‘Feng Dan’ genome, and the coding DNA sequences (CDS) were retrieved to design RT-PCR primers using Primer 5 software (http://www.premierbiosoft.com/). The normalization utilized the endogenous reference gene *PoPUF1639*. Details of the primer sequences can be found in **Table 3**.

**Table 3:** *PoSOS1* genes Primers.

Name	Sequence (5’-3’)
*Pos.gene4502.mRNA-1-F*	ATGGAGACAGGTAGAGACATAC
*Pos.gene4502.mRNA-1-R*	AACTGCAGCAACAGAAGAGG
*Pos.gene39601.mRNA-1-F*	TCGGACAACCCAAGATTGTC
*Pos.gene39601.mRNA-1-R*	GTGAAGGCTTTGATACCTGTG
*Pos.gene64207.mRNA-1-F*	GCCACTGTTACGCTTCTTTC
*Pos.gene64207.mRNA-1-R*	AAGCTTCCAGCATCAGTGTC
*Pos.gene68372.mRNA-1-F*	ATGAATGAAGAAGAAGCTTCCC
*Pos.gene68372.mRNA-1-R*	CAACTCCAAATTCCGCGATG
*Pos.gene10758.mRNA-1-F*	TCAGATCATGATGCTCGTGC
*Pos.gene10758.mRNA-1-R*	AGCAAATGTCACTATAGCTCC
*Pos.gene1017.mRNA-1-F*	CTGAATCGGATCGGTTTGAAG
*Pos.gene1017.mRNA-1-R*	ACTGCCTGTTGATCAGCAAC
*PoPUF1639-F*	AAACGAGTCGGTTGAAGATGAG
*PoPUF1639-R*	TATGCGGTGGATTTCGGAG

### 4.10 Quantitative real-time PCR assays

The TaKaRa MiniBEST Plant RNA Extraction kit (9769, Takara) extracted total RNA from leaf samples following the product instructions. The concentration and purity of RNA were measured using a Thermo Scientific™ NanoDrop™ spectrophotometer. First-strand cDNA was synthesized from 1 μg of total RNA utilizing the PrimeScript™ RT Reagent Kit (RR047A, Takara) in a Life Technologies PCR machine. Quantitative PCR was conducted using a Longgene CFX96 Real-Time System with the TB Green™ Premix Ex Taq™ II Kit (CN830B, Takara). The reactions comprised 2 μL of diluted cDNA template in 20 μL, with three technical duplicates for each sample. Thermal cycling parameters adhered to manufacturer standards. Threshold cycle (Ct) values were evaluated with the 2^ ^(-ΔΔCt)^ methodology.

### 4.11 Statistical analysis

The statistical program GraphPad Prism 9 (https://www.graphpad.com) was used to create the graphs, and the data was analyzed using one-way ANOVA in SPSS version 13.0. The results were displayed as the three replicates’ mean SD (standard deviation).

## 5 Conclusions

In conclusion, this study provides a comprehensive genome-wide identification and characterization of the *SOS1* gene family in *Paeonia ostii*. Our results reveal the complexity and diversity of the *SOS1* gene family in *P. ostii* and provide new insights into their evolutionary relationships, gene structure, and expression patterns under salt stress conditions. Identifying differentially expressed *SOS1* genes under salt stress treatments suggests their potential role in salt stress tolerance in *P. ostii*. This study lays the foundation for further research on the functional characterization of *SOS1* genes in *P. ostii*. It provides valuable resources for breeding programs to improve salt stress tolerance in *P. ostii*. Furthermore, our findings contribute to a better understanding of the molecular mechanisms underlying salt stress tolerance in plants and may have implications for developing salt-tolerant crops.

## Data Availability

The datasets presented in this study can be found in online repositories. The names of the repository/repositories and accession number(s) can be found in the article/**Supplementary Material**.

## Supplementary material

Supplementary Figure 1 Multiple SOS1 protein sequence alignment.
