# Supplementary material for: Genome-wide identification, characterization, and expression analysis of the SOS1 gene family in the medicinal plant Paeonia ostii under salt stress
Source: Front Plant Sci. 2025 Jul 30;16:1614011. doi: 10.3389/fpls.2025.1614011 (PMC12344307; doi:10.3389/fpls.2025.1614011)
Supplement: Supplementary Figure 1 — Multiple SOS1 protein sequence alignment. [file DataSheet1.pdf]

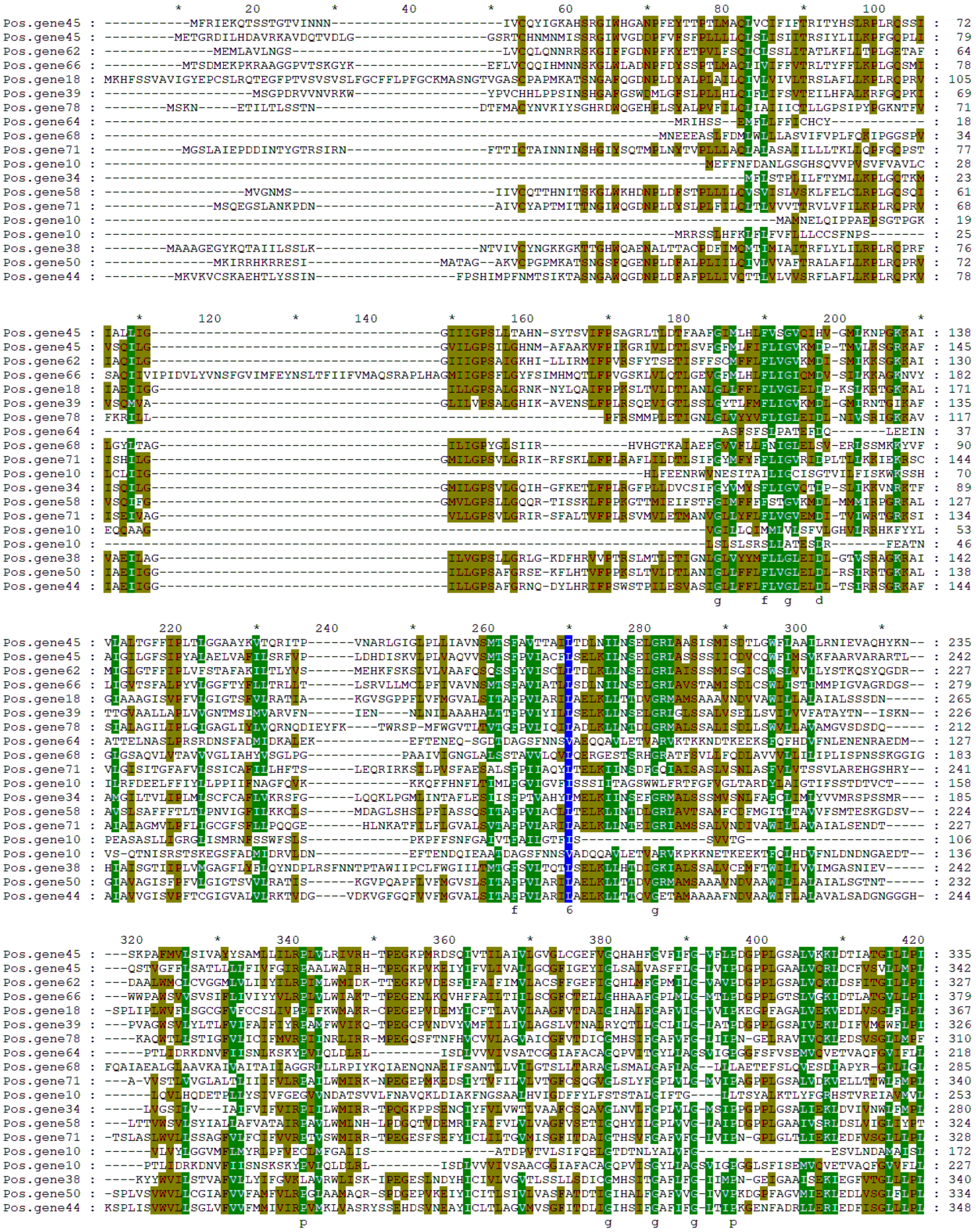

```
                         *       440          *       460          *       480          *       500          *       520
Pos.gene45 : FCAMSGLRTNVHSLVDLKTSL----IAEVIILVGYMGKFTGTLLPSLCF--------------KIPISDAVSIALLMSCKGIIDINIYCLWLDIE---------- : 412
Pos.gene45 : FFTTCGLKMDVFTIQNLKNVG----VILLVVYVAFLGKIIGTILLPLFW--------------RMPFRDAISIALIMNSKGIVELALLNDLERVN---------- : 419
Pos.gene62 : FFSVSGAKVDLS-VVDMKYFG----IVQLLSVINFSGKLIGTMLPSLYC--------------KMPALDAFCLGLIMSAQGITDILILENALHLY---------- : 403
Pos.gene66 : FFAISGLKTNLFSLGQVKSAL----ITEFIIIMGYLGKFMGTLVGGIFC--------------HLPFWDAFSIALIMSCKGIIEVATYIIWKLGQ---------- : 456
Pos.gene18 : YFVSSGLKTNVATIKGV-QSWG---LLVLVIFTACLGKIVGTVAVSLGC--------------KMPFQEAIAIGFLMNSKGLVELIVLNIGKERK---------- : 444
Pos.gene39 : YITTCALRADLISLFVIHENARMLWLNTILIILMFLVKIVATMIPLLCC--------------KVPFYDAFTIALIISCKGVVELATYSWFRDSE---------- : 407
Pos.gene78 : FFAIVGIRTNLGSIRFQGTSWF---KVAGVVAGACLAKIMSTILVAFLFGIRIQAKGGGAFFNNIQLRESIALGVLMNTKGLLALVTLYVARDRL---------- : 402
Pos.gene64 : FALGLEFSATKLRVVRAVAVLG---GLLQIFLFMCLCGVTATLCGGKPS-------------------EGVFVGAFLSMSSTAVVLKFLMERNSIN--------- : 292
Pos.gene68 : FFMTVGMSIIPKLILSNFPVIMG-----TLGLLIAGKTIIVALVGKVFG----------------ISIISAIRVGLLLAPGGEFAFVAFGEAVNQG---------- : 360
Pos.gene71 : YFVKNGLVINIFGVK-QKNYV----IVQSLILVTCAGKFLGALLPSLYF--------------RMPMKDAIALGLVMNVQGVLELGMFKMMKRK----------- : 415
Pos.gene10 : MAYLSYMLAELFELSG------------ILTVFFCGILMSHYAWHNVTD-----------------SSRITTRHVFATMSFIAETFIFLYVGMD---------- : 318
Pos.gene34 : FFLTNGMAMNVFSVK-PKTCG----AVQLIIILGGCVGKFLGSFLPSLFW--------------KMPVKDAIALGLVMNTQGLLELGIFRMMKLRKVCLEFIFVRI : 366
Pos.gene58 : FLAVSGLKTNIF-EIHLRAFW----VVGIVVVFACLVKIGAVILPAMYT--------------GLSTREAIVIGLVINAKGINELVIYNLWLQGK---------- : 400
Pos.gene71 : FFAISGLKTDISSIKGA-ATWG---TLFIVIVLGCLGKIAGTVLVTFFY--------------KMPLREGVTIGLLMNTKGLVEMIVLNVGKDQK---------- : 405
Pos.gene10 : YRTMSLVRSHASSGQ---------------NFFMVVVRFLETFVGSMYM----------------LAEGIGISGIVSILFTGMVMKHYTFSNLS---------- : 235
Pos.gene10 : FALGLEFSMSKLRVVRAVAVLG---GLLQIFIFMFLCGITASICGGSPS-------------------EGVFVGVFLSMSSTAVVLKFLMEKNSAN--------- : 301
Pos.gene38 : FFALIGFRANFNFLQDK-SRWL---VTGITVVGLCLAKILSTILVTTFY--------------NMRLREGIALGVLMNTKNLLALVILYMGRDRL---------- : 417
Pos.gene50 : YFVSSGLKTNVATISGG-QSWG---LLVLVIFTACFGKILGTLVVSMIF--------------KVPAREAIAIGFLMNTKGLVELIVLNIGKDRK---------- : 411
Pos.gene44 : YFASSGLKTDVAKIRGL-EAWG---LLGLVITTACAGKILGTFAVAMLC--------------KLPARESITLGILMNTKGLVELIVLNIGKEKR---------- : 425

                *       540          *       560          *       580          *       600          *       620          *
Pos.gene45 : -------------------------------------------------------IADQCLFTLLVITMVVMTGIATPIASYLYDPSKRYKAYQGRTIYRVS--- : 459
Pos.gene45 : -------------------------------------------------------VMNEECFAIMILSVVVMTGVISPLVKTLYDPSRRFLAYKKRTVLDS---- : 465
Pos.gene62 : -------------------------------------------------------IIDEQSYTVIVISMVLTTGAITPIVKYLYKPSRRYKSYKRRTILHA---- : 449
Pos.gene66 : -------------------------------------------------------ILNSQSFSLLLITMLLVTGVARPIIGHLYDPSRRYMTYTRRTIQHS---- : 502
Pos.gene18 : -------------------------------------------------------VLNDQTFAIMVIMALFTTFITTPLVVAVYKPAKRMGKAYKQRTIE----R : 490
Pos.gene39 : -------------------------------------------------------NIDTDTFTMMVIVTLLSASVVPTLVKLLYHPSRKYAGYQKRSIMHT---- : 453
Pos.gene78 : -------------------------------------------------------VFNDQACAIMMIAILIMTILVGPFTLAIFRPSKHYRQHTHSRSIL----R : 448
Pos.gene64 : -------------------------------------------------------TIHGQVTVGTLILQDCAVGLLFALIPIIGGTSGVLQGVISMTKSLV---- : 338
Pos.gene68 : --------------------------------------------------------IMSPQLSSLIFLVVGISMAITPWIAAGG------------------ : 388
Pos.gene71 : ----------------------NS-------------------------------IDTEAFVVMCTSMLVVTMAVTPIVRYLYDPSRKYRVYRRRTVMHL---- : 462
Pos.gene10 : -------------------------------------------------------ALDIEKWKMSKIRGITSGIQIVAVTGAAGGDQATGDGGIRPSNLGG---- : 364
Pos.gene34 : SAQLGPHGLAQWVRQSDLGVAVNSGVPSSNLTTASCWTYMPWSKGMLRAEGLGCWAIIDAEICTIMIISMVIVTGVITPIVKYLYDPSRRYIVYNKRTIMHS---- : 467
Pos.gene58 : -------------------------------------------------------LIENSEFTIVVISVLVVTAIITPLIRILYDPSKQYVSIKRTSIQQA---- : 446
Pos.gene71 : -------------------------------------------------------VLDNRAFAIMVIVAVIMTGIITPIVTTIYRPAR-RFIPYKRRTIQ----R : 450
Pos.gene10 : --------------------------------------------------------ENSQRFASAFFHLISSLAETFVFIYMGFDIAMEKHSWS---------- : 273
Pos.gene10 : -------------------------------------------------------AIHGQVIIGTLILQDCAVGLLFALIPVIGGTSGVLQGVYSMIKLLV---- : 347
Pos.gene38 : -------------------------------------------------------VLDDIPVAVMILSVLVMSLLVMPITLGIYRPSRRFMQHKY-KTIL----K : 462
Pos.gene50 : -------------------------------------------------------VLNDQTFAVLVIMALFTTFITTPIVMAVYKPAR-KGSPYKHRTVQ----R : 456
Pos.gene44 : -------------------------------------------------------VLNDETFAILVIMALFTTFMATPTVMAIYKPTR-GSTSFTHRKLGDFSIT : 474

                    640          *       660          *       680          *       700          *       720          *
Pos.gene45 : -NRDLHLRMIVCIHNEHNVSP-MINLVEASNPTRINP-ITIHVLQIMEITGRSTAILVPHHQKN------KPSKDMTHSEHIVNAFKLVEKHSHGNVMVQHFTAI : 555
Pos.gene45 : -KHNEELRVIVCIHSQDNVQE-VVNLIDASRPTKESP-INLVVLHIVKIEGRASSLLIPHRPRE------KPSINPTQSERIFNAFRKFEQEYRNLVTIHCFKGI : 561
Pos.gene62 : -KPDAELRIIACIYHEDNTPS-IINIFEVSNPTVENP-ICFYVAHILDIDGRSTPLLVTHRPGD------KNSIQSSNSDHIINAFRIYEQQNEGAVTINLFTSI : 545
Pos.gene66 : -KENVELRIIVCIHHEDNVPT-MVNLLEASNPTRFNA-ISILVIQIMEIIGSATAVLVPHGQQN------KQNFNLAHSEHIVNAFNHYEKQNHGRVSVQHYTAI : 598
Pos.gene18 : KDTNTQLRIMTCFHSTRNIPA-MLNLIEASRGREKKGGLCVYAMHIMEISERSSAILMVHKARKNGLPFWNKGQSND-SNQVIVAFEAF-SQLS-KVSIRPMTAI : 591
Pos.gene39 : -KPNSELRIVACIHGEDNTSSRIINLIDASCPTRQNP-LAVYVLHLIEIVGRATPIFISHQMQK------KFISNKSYSKDVIHDFAKYERENQGAVTVHAFTAI : 550
Pos.gene78 : LKPDAHLRIIPCFHDSRNVQG-IINLIEASNPTMRSH-VFVFALYLIEIVGRASAMFAVCTKRKPHDMYKNPRYGETDLDHITNALQNYGHKRCRAVSVQTHSVV : 551
Pos.gene64 : --ILITFLVILSILARTCVPWFLKLMISLSSET----------NELYQIVSVAFCLLVAWCSDKLG------------LSLEIGSFAAGVMISTTDIGQHTLEQV : 419
Pos.gene68 : -------QLIASRFELHDMRS----LLPVESETDDLQ-------------------------------------------DHIIILCGFGRVGQIIGQLLSER----L : 437
Pos.gene71 : -RPHSELQVIVCIHDQENVPA-TINLIEALYPTVHNP-MAIYVLHITEIIGRADPLLIPHKLNK------KPSSKASPSERIFNAFRYYEQSNHGFVSVHPFEAI : 558
Pos.gene10 : --FEYPAKGCPTVGRPQPEVAGARRQGSLNSLQQPSP------RRRKNRPAEGAVIEVDFGEGE---------------DEVEGGFDGNEEWEE----RRRGWCL : 442
Pos.gene34 : -KSDVELRIIACIHGQENVSA-TINLIEAFSPTKQRP-MAIYVLHIMEIIGRANPLLIFHRVNK------KESFKSLLSDPIVNAFRLFEQNNQGFVSVHPFTSI : 563
Pos.gene58 : -KCNTEFRIMVCIQNQESVPT-IINILEVSYATEESP-VAVIALHLIQIVGRTTPMLVAHQPERT----LDKKLSLSRSNHLINALRHYEQHNEGAVSIQSFTSM : 544
Pos.gene71 : SKPDAELRVIVCIHTPRNVPT-IINLLEASNPTKKSP-ICVYVLHLVEITGRASAMLIVHNTRKSGRPALNRTQAQS--DHIINAFENY-EQHAGCVSVQPLTAI : 550
Pos.gene10 : -------HIFIGVARAANVFS-CAYVVNLIRPAR-------------------------------------------------RQIPLKHQKALWYSGLRGAMAFALAL : 325
Pos.gene10 : --VLILFLAVLSILCRTCVPWSLKLMISLSSQS----------NEIYQIASVAFCLLVAWCSDKLG------------LSLEIGSFAAGVMISTTDIAKHTLEQV : 428
Pos.gene38 : AKPDAELRIIACIYESPNVQG-VINLLETANPTKQSP-LSLIALHLIEITGRASAMLAVHTGRKSSTLN--PTREEAESYKITTAFENYGEE-STAVSVEMLTVV : 562
Pos.gene50 : KDPDSELRIIACFHSTRNIPT-MINLIESSRGIRKRGRLCVYAMHIMEISERSSAISMVHKARNNGLPFWNK-KRDD-RDQMVIAFEAF-QQLS-SVSVRPMTAI : 556
Pos.gene44 : DNPKDELRIIACVHGPGNISS-FTSLIESTQSTTKSQIVKLYLMHIVEITERSSSIKMVQRFRKNGLPFINRFRRGGIQDRVAAAFQAY-SQLG-RVLVRPTTVI : 576
                                                              l  l                                      f                 6

                 740          *       760          *       780          *       800          *       820          *       840
Pos.gene45 : SPYASMHDDICNLAIDKKISIIILPFHKHWAIDGT---IETSFHFIKSVNENVINNAPCSVGVLVDRS--PIGG-N-------------------QSLLSYDSTY : 635
Pos.gene45 : SPYATMHNDVCLLALEKRTTLIIIPFHKQWTKGER---VES-SYAFRHLNKNVIDKAPCSVGILINHK-NQRKHRH-------------------AVSEP--SFY : 640
Pos.gene62 : SPYVTMHEDVCTLALEKKTSMVIIPFHRRWTIQG----TEESANIIRAVNRSIIRKSPCSVGVYVDRG--TLRGTN-------------------SSTMSSQSTY : 625
Pos.gene66 : APYASMHDDICTLALDNKITIIIVPFHKKWTIDGS---IGATLPNIRTVNRNVITRAPCSVGVLVDRG--HMGD-H-------------------PSILSGQSLY : 678
Pos.gene18 : SHMSDIHEDICTSAESKRAAMIILPFHKHQRVDG---TLETTRNEFRWVNRRVIEHAPCSVGILIDRGLGGTTHVP-----------------------ASNVSS : 670
Pos.gene39 : SPSILMHEDICTLALNKLTSLIIIPFHRKWTLQGKS--IEAQERYLRTLNRIVIDLAPCSVAIFIDRG------------------------------HSRKTSKLEY : 626
Pos.gene78 : SPYPTMDIDICNLAEEKGITFIILPFHKQQTVNR---KMKDINPEFLGVNQNVIAKARCSVGIFIDRGFSTSLSMEPQSSMSSFMELWSSTSSSFIESRSSNFNC : 653
Pos.gene64 : EPIRNFFAAIFLASIG-----MLIHVHFLWNHVD----------IILAAVILVIIIKTIVVAIVVKG--------------------------------------FGY : 474
Pos.gene68 : IPFVAIDVRSERVAVGRALDIPVFFGDAAITLDTP----GANYRTVWAISKYFPNVKTFVRAHDVDHG------------------------------------- : 501
Pos.gene71 : SSYATMHDNVCSIALDRRVSLVIVPYHKKFAKGAT---METSSNGIKIMNYNVIDNAPCSIAILVDR--GLIKTPT-------------------PILQSWSDSY : 639
Pos.gene10 : DLAEWVVEEEGNRGRKGWPTIFWIGFGTLMAICST-------IILLILIGRAAFVFPLSAISNYMNRRNAETSG-------------------------SITIKH : 515
Pos.gene34 : STYPTMHDDVCTIARDKRVSLVIIPYHKGIAANGA---IESFKKGHEIVNYNVIDKAPCSIGILVDRDRGLLNNSK-------------------PILMG-AALY : 645
Pos.gene58 : SHFETIHDDICRVARDEKATIVIVPFHKQWAIDGS---VGMVNRPIQNVNLNVIDKAPCSVGILVDRG--ILSG-S-------------------LSVLNGRSIY : 624
Pos.gene71 : SPYSTMHEDICNLAEDKRVAFVIIPFHKQQTVDG---GMEATNPAFRMVNQNVIANSPCSVGILVDRGLSGSTRLA-----------------------ANQISH : 629
Pos.gene10 : QSVHDIPEGHGQTIFTATTAIVVITVLLIGGTTG----------TMLEALHVVGDGNDGPIGESFEGN------------------------------------NGY : 386
Pos.gene10 : EPIRNFFAAIFLSSIG-----MLIDAHFLWNHVD----------ILLASVMLVIIIKTIIITMVVKG--------------------------------------FGY : 483
Pos.gene38 : SPYTTMDEDICSLAVDKYVNFIILPFHKQRTSDG---KMRETKPDVLGVNQNVFANAPCSAGIFIDRGLGAAELMG----------------------ESGEFKC : 642
Pos.gene50 : SAFNSIHEDICTSAHQKRAAMILLPFHKHQRVDG---TMESIGHSLRLANERVIRHAPCSVGILVDRGLGGTTQVS-----------------------ASDVSY : 635
Pos.gene44 : SPLTTMHEDICHVAEDKGVTMIILPFHKQWRGEGDEQTVDNMGHGWRAVNQKVITNASCSVGVLIDRGFGSTTQQTP---------------------NPSSTVRQ : 661
                             a               h
```

```
                      *        860         *        880         *        900         *        920         *        940
Pos.gene45 : RIAILYLGGGDDVEAIAYSRRMVEHPN-IILTVVWIEQ-------------------------PGCR--------LDIGIMNDFTASVGNNKRVTYKKEMVKDGE : 706
Pos.gene45 : HVVVLFFGGADDREAIAYARRMLIHQN-VKLTLLRFCA------------------------YKEIVGGTARSRSLDSELIEEFRRNHMRDERVSYQEKAVKSKI : 720
Pos.gene62 : NIGMFFLGGKDDREAIAYAMRMSQHPD-VSLTVIRFLE-------------------------STRRGEYNLERELDFDVTTDFKHSTAGNKRHLYREEVVKDSV : 704
Pos.gene66 : CIALFFLGGPDDREAIAYAKRMGEHQH-VRLTVVWFKP-------------------------KSEHESK--EKGSDRVAIDEFKSSSVGKSRIVYKEETVIDGV : 755
Pos.gene18 : LVTVLFFGGRDDREAIAFGARMAEHPG-ISLLVVRFLLDPESVGEFVEVNIKDSGISSNSIHPVGKSVN-------DEEFLADIQKKVSSDGSIKYEERVVRNVG : 767
Pos.gene39 : SVAVLFFGGQDDREAICYAKRMATVPNNICVTVVQFVAS------------------------GDEAATVSWEKMLDSSLFNELKHRNKDDIHINFVEKVVDGGP : 707
Pos.gene78 : RITVLFVLGCDDREAISYAWRMASHPG-VSLDVIRFIPGEDFVN-------NNKDESLNVLTNYEREKK------LDDTYIKEFKLKTKMDEAITYSEKVVNSGV : 744
Pos.gene64 : NNKTSLLVCMSLACIGEFAFVILSRAS--NIHLVEVSA-----------------------------SVHANSPLLVVSCLGVIRSLSSLLQGKLYLLLIGTTAL : 548
Pos.gene68 : ------VVPETLEPSIQLAAAVLAQAK---LPTSEIAS-----------------------------------------TINEFRSRHLSELTELCESSGSSLGY : 556
Pos.gene71 : RVAVLFLGGADDREAIALGVRMASHRN-INLTLFRLLDNC--------------------KNIDTKDDTEKRKRMLDHELVTEFRHKMVGNYRIKYIEEVVVDIT : 723
Pos.gene10 : QIIIWWAGLMRGAVSIALAFKQFTNSG-VTWDPVNATMITN---------------------------------TIVVVLFTTLVFGFLTKPLINYLLPHNANNN : 586
Pos.gene34 : RIGVLFLGGADDREAIAIGTRMASHPN-ISLTVIRLLE-----------------------DGDTSTDG-MGERGLDDELVTDFRLNTAGNHRVMYIEEVVMNGL : 725
Pos.gene58 : HVAVIFIGGGDDVESIAYGTRMSRHGS-VNLTVIRFLL-------------------------FGSENTR--ERKRDTDLIDEFRQAIGNNGGFLYKEEVVRDGV : 701
Pos.gene71 : HIVVLFFGGPDDREAISYAWRMSEHPG-ISLTVMRFIPGEDAADTVVEPGHDPNDPRVLTVVTDTEREK-----QLDEDYINEFRMKNVNEESVVYTEKVVNNGE : 728
Pos.gene10 : IAPSYDEGGSSGNTTLKMKLKEFHKST------ASFTA-------------------------------------FDKNYLTPFFTSQNGDDEEEFDEPVPSSRR : 448
Pos.gene10 : TNKASLLVCMSLACIGEFAFVILSRAS--NIHIVEG---------------------------------------------------------KLYLLLIGTTAL : 529
Pos.gene38 : RIAVLFVVGRDDREAIAFAWRMAGRPG-VTLEVTRFIPGENVADVESMDLPDSTDGILSAMTNHEREKQ------VDDAFIKGFRVKTLSNDSMMYTEKVVNSGA : 740
Pos.gene50 : AVVVPFFGGGDDREAISYGVRIAEHPG-ISVTVIKFVPPPGKSLMMDQGSGKAAAGAVGIDDMASDGVN-------DENFISDIISNKKE--SLIYEEKVVSSKE : 730
Pos.gene44 : RVCIVFFGGPDDRETFELGGRMAEHPT-VKVTIVRFIEKSGMKSNRLVLRPSPTKGRDNNYCFSTAAMNRDKEKELDEAAVAKFRSRWEG--SANYEEKVESNIV : 763
                     g                                                                   d

                 *        960         *        980         *       1000         *       1020         *       1040         *
Pos.gene45 : GTVQVVAS-MEDDIDIFIVGKNHEPNSPATLGLAEWRQNKFSELGIIGDMIAISD--YTFSVLVVQQPLGAD-----------------FVDCPKGSHGYKK--- : 788
Pos.gene45 : DVLSVCRSVLGTDYNIVMVGRRHGE-SQFMCELARWN-ERG-ELGTVGEIIAASVTKSGVSVLVVQQQTRVWG-------------------MHDPEEPTHLIKL : 803
Pos.gene62 : EMVSIIHE-VENSLDIVIVGRSHASDSLLLVGLTEWN--EFPELGLIGDMIASSDSNCHVSVLVVQQEAFDGEDPILQSPKYIMEDSVAVVDMPRDNSKIWPHKF : 806
Pos.gene66 : GTTRVIRT-MEDSFDIVLVGKNHDPDSQPLLGLTEWS--ECPELGVIGDMIATSD--FRFSVLVVQQQPL----------------------------------- : 820
Pos.gene18 : ETIEVVKE-FGRRCNIFLVG--RMPEGQIVAALKGES--EFQAIGPVGNLIASTEISTPASVLVVRQYHGALVLSNEVELPERVRTNWIEYCYFLWT-------- : 859
Pos.gene39 : QMAKMIRS-MTHSYDIMIVGRGHDVECPQLTGLIDWC--EFRELGVVGDIIAADDINIKSSVLVVQQQKQFSG-------------------------------- : 777
Pos.gene78 : ETIGKIKS-MKHNCDIYIVGRGQGAETPLTKGLSDWT--DNPELGAIGDLIVSSEFATPVSVLVVQQYTAGYVN--DVLDISEGSSHRREPLNSFGHMTWRLPVE : 844
Pos.gene64 : SLIGFKGDILRADSAKRIALMILDFGIKLLLQFTFVN--ILKTLIFYWGLITINTYLDWVVSKIILYK------------------------------------- : 614
Pos.gene68 : GYSRVMSK------------------------------------PKPQPTDSSDDNQIAEGTIAI---------------------------------------- : 585
Pos.gene71 : GTISVIRS-LENNYELIIVGRHHDNKSPLTLGLRDWD-EDT-YLGVIGDFLTSDDFKGNNTVLVVQQHTNVVDESKENQ---IVQKFPMFQNMGEEETEDTPIQR : 822
Pos.gene10 : HHHPESRS-PKEDVTLPLLSFEESAATNILRAKNSLTMLIERPVYTIHSYWRRFDDTYMRPIFGGPVSNQPLC-------------------------------- : 658
Pos.gene34 : GTLTVIKS-MENNYELIMVGKHHDKTSPFTAGLVGWN-EGTTYLGLIGEAFTSEDFKSNNTVLVVQQHTYQGNECHENQKEKLLQTIKMGRDMVDEEAEDTPMQK : 828
Pos.gene58 : GLATLLRG-LEDCFDIVIVGR-HHPESPLLSGLDQWS--ECQELGVIGDMIASPDFGSTASVLVIQQQRLKGGNIISTTPTRLKQQPILDDRDPFLHDDVRN--- : 799
Pos.gene71 : ETVAVIRS-MENVHDIYVVGRGQGMVSPLTAGLTDWS--ECPELGAIGDLIASSDFASTVSVLVVQQYVGVGPQGDGVGTPDSPGQSEEQSFSMQQMNRRPQPPR : 830
Pos.gene10 : GEFRGRG-------------------------------------------------------------------------------------------------- : 455
Pos.gene10 : SLVTTP-------LLFKLIPAVINFG--VLLRWFSPD--SQIEVAFKGDIIHSDSAKQRISLIIQGSHD------------------------------------ : 587
Pos.gene38 : ETVAAMRE-MNHDYDIYVVGRGHGVVSPLTAGLSEWT--DNPELGAVGDIIVSSEFAASVSVLVVQQFAGAEYDKENMPGMINGLPGHKEQYNTS-------LED : 835
Pos.gene50 : EIIEALKL-KN-RCNIFLVG--RMPP---TVALMEKS--DCPELGPVGSYIASSEFSTSASVLVVQQYNPFANLYPLVEEQNPDSTDMTEPDTPV---------- : 816
Pos.gene44 : EEVLTIGR-SG-DYDIVVVGKGRFPSTMVAELVDRQV--EHTELGPIGDVIASSGHGIVSSVLVVQQHDLSHAEETPVSKVLHREDAIITVLGSSSSI------- : 857
                            l                               g

                    1060        *
Pos.gene45 : -------------------------- :   -
Pos.gene45 : -------------------------- :   -
Pos.gene62 : IFLQVTLQLEGVSTSSAVFSKLTSER : 832
Pos.gene66 : -------------------------- :   -
Pos.gene18 : -------------------------- :   -
Pos.gene39 : -------------------------- :   -
Pos.gene78 : STGAFEPSVHCRGI------------ : 858
Pos.gene64 : -------------------------- :   -
Pos.gene68 : -------------------------- :   -
Pos.gene71 : NVSLSS-------------------- : 828
Pos.gene10 : -------------------------- :   -
Pos.gene34 : K------------------------- : 829
Pos.gene58 : --------VEGARDQNASWTISINRY : 817
Pos.gene71 : GQPVFSP------------------- : 837
Pos.gene10 : -------------------------- :   -
Pos.gene10 : -------------------------- :   -
Pos.gene38 : DNGGRKPFVHHSGY------------ : 849
Pos.gene50 : -------------------------- :   -
Pos.gene44 : -------------------------- :   -
```
